# Overexpression of ESYT3 improves radioimmune responses through activating cGAS-STING pathway in lung adenocarcinoma

**DOI:** 10.1186/s40164-024-00546-y

**Published:** 2024-08-05

**Authors:** Zan Luo, Ying Li, Bin Xu, Tenghua Yu, Mingming Luo, PeiMeng You, Xing Niu, Junyu Li

**Affiliations:** 1 Department of Radiation Oncology, The Second Affiliated Hospital of Nanchang Medical College), Jiangxi Cancer Hospital, Nanchang, Jiangxi 330029 China; 2 Jiangxi Key Laboratory of Oncology, The Second Affiliated Hospital of Nanchang Medical College), Jiangxi Cancer Hospital, Nanchang, Jiangxi 330029 China; 3 Department of Radiation Oncology, Hubei Cancer Hospital, Tongji Medical College, Huazhong University of Science and Technology, Wuhan, 430079 Hubei China; 4 Laboratory of Tumor Metastasis, The Second Affiliated Hospital of Nanchang Medical College), Jiangxi Health Committee Key (JHCK), Jiangxi Cancer Hospital, Nanchang, Jiangxi 330029 China; 5 Department of Breast Surgery, The Second Affiliated Hospital of Nanchang Medical College), Jiangxi Cancer Hospital, Nanchang, Jiangxi 330029 China; 6 Jiangxi Clinical Research Center for Cancer, The Second Affiliated Hospital of Nanchang Medical College), Jiangxi Cancer Hospital, Nanchang, Jiangxi 330029 China; 7 Department of Radiation Oncology, Jiangxi Key Laboratory of Translational Cancer Research, Cancer Hospital of Nanchang University, Jiangxi Cancer Hospital of Nanchang University), Nanchang, Jiangxi 330029 China; 8 Experimental Center of BIOQGene, YuanDong International Academy Of Life Sciences, Hong Kong, Hong Kong 999077 China; 9 China Medical University, Shenyang, Liaoning 110122 China

**Keywords:** Radioimmune responses, ESYT3, cGAS-STING pathway, DNA damage, Lung adenocarcinoma, Type I IFNs

## Abstract

**Background:**

Radiotherapy can modulate systemic antitumor immunity, while immune status in the tumor microenvironment also influences the efficacy of radiotherapy, but relevant molecular mechanisms are poorly understood in lung adenocarcinoma (LUAD).

**Methods:**

In this study, we innovatively proposed a radiotherapy response classification for LUAD, and discovered ESYT3 served as a tumor suppressor and radioimmune response sensitizer. ESYT3 expression was measured both in radioresistant and radiosensitive LUAD tissues and cells. The influence of ESYT3 on radiotherapy sensitivity and resistance was then investigated. Interaction between ESYT3 and STING was evaluated through multiple immunofluorescent staining and coimmunoprecipitation, and downstream molecules were further analyzed. In vivo models were constructed to assess the combination treatment efficacy of ESYT3 overexpression with radiotherapy.

**Results:**

We found that radioresistant subtype presented immunosuppressive state and activation of DNA damage repair pathways than radiosensitive subtype. ESYT3 expression was remarkably attenuated both in radioresistant LUAD tissues and cells. Clinically, low ESYT3 expression was linked with radioresistance. Overexpression of ESYT3 enabled to alleviate radioresistance, and sensitize LUAD cells to DNA damage induced by irradiation. Mechanically, ESYT3 directly interacted with STING, and activated cGAS-STING signaling, subsequently increasing the generation of type I IFNs as well as downstream chemokines CCL5 and CXCL10, thus improving radioimmune responses. The combination treatment of ESYT3 overexpression with radiotherapy had a synergistic anticancer effect in vitro and in vivo.

**Conclusions:**

In summary, low ESYT3 expression confers resistance to radiotherapy in LUAD, and its overexpression can improve radioimmune responses through activating cGAS-STING-dependent pathway, thus providing an alternative combination therapeutic strategy for LUAD patients.

**Supplementary Information:**

The online version contains supplementary material available at 10.1186/s40164-024-00546-y.

## Background

Lung cancer is one of the major causes of cancer-related deaths globally [1]. Non-small cell lung cancer (NSCLC) accounts for 85% of all lung cancer cases, of which lung adenocarcinoma (LUAD) represents the dominating histopathological subtype (~ 60% of NSCLC cases among Asian patients) [2]. Radiotherapy is an important modality in LUAD therapy [3]. Radioresistance to primary tumors and metachronous metastasis is still the major cause of therapeutic failure for patients with locally advanced LUAD [4]. Thus, it is a priority to identify reliable targets for individualized radiosensitization regimens.

Immune checkpoint blockade (ICB), e.g., anti-programmed death 1 (PD-1)/programmed death ligand 1 (PD-L1), has emerged as a promising treatment choice against advanced NSCLC [5–7]. Nonetheless, ~ 80% of patients cannot benefit from ICB alone due to intrinsic or acquired resistance [8]. Although many clinical trials have been conducted, nearly no immune-oncology agent or combined therapy has exhibited activity in the refractory setting. Radiotherapy has been utilized as a combination of immunotherapy for further enhancing therapeutic effects [9–11]. In an anti-PD-1 resistant murine model of NSCLC, tyrosine phosphatase SHP-2 and PD-L1 inhibitors in combination with radiotherapy can enhance systemic antitumor effects [12]. In vitro and in vivo assays demonstrated that radiotherapy plus oxidative phosphorylation inhibition (IACS-010759) overcome PD-1 resistance as well as heighten antitumor immunity in NSCLC [13]. A multicentric, retrospective cohort showed that immunotherapy combined with stereotactic radiotherapy is correlated to more favorable intracranial local progression-free survival for NSCLC patients with brain metastasis [14]. Thus, in-depth exploration of the molecular mechanisms underlying radioimmune responses is required for providing the theoretical basis for combination therapeutic regimens.

In this work, we proposed a novel radiotherapy response classification for LUAD, and unveiled a potential ESYT3-mediated radiotherapy resistance mechanism. Especially, we discovered ESYT3 as a tumor suppressor and a novel radioimmune response sensitizer for LUAD, which can activate cGAS-STING-dependent DNA damage response through directly interacting with STING, acting as a potential treatment target for improving LUAD prognosis.

## Materials and methods

### Acquisition of LUAD multi-omics data

The transcriptome data and clinical information of four LUAD cohorts: TCGA-LUAD (*n* = 502), GSE31210 (*n* = 226) [15], GSE72094 (*n* = 442) [16], and GSE30219 (*n* = 293) [17] were collected from public databases. Among them, the TCGA-LUAD was set as the training cohort, with others as the validation cohorts. The frequency of single nucleotide variations (SNVs)/copy number variations (CNVs) across TCGA-LUAD individuals was obtained and estimated based upon the Gene Set Cancer Analysis (GSCA) platform [18] or matfools package [19]. Tumor mutation burden (TMB) of TCGA-LUAD samples was also assessed.

### Radiotherapy response subtype analysis

A previous study identified differentially expressed genes (DEGs) in radioresistant LUAD cell line (A549RR) versus its parent cell line (A549), which were regarded as LUAD radioresistance-related genes (RRGs) (Supplementary Table 1) [20]. DEGs between LUAD (*n* = 502) and normal lung tissues (*n* = 59) were selected utilizing limma package [21] with the criteria of |log2fold change (FC)|>1 & adjusted *p* < 0.05. Next, DERRGs were determined via intersecting the DEGs and RRGs. Univariate-cox regression method was implemented on DERRGs with LUAD survival. Prognostic DERRGs were retained for consensus clustering utilizing ConsensusClusterPlus tool [22]. According to the consensus matrix, LUAD patients were classified as radioresistant and radiosensitive subtypes. Transcriptome difference between subtypes was proven via principal component analysis (PCA). The difference in overall survival (OS) and stage between subtypes was evaluated. The top 100 up-regulated markers between subtypes were selected via limma package. Based upon them, to assess repeatability and accuracy of molecular subtypes, nearest template prediction (NTP) approach [23] was carried out both in the GSE31210 and GSE72094 datasets utilizing CMScaller package [24].

### Functional enrichment analysis

The gene sets of DNA damage repair, X-ray, and UV responses, and reactive oxygen species (ROS) processes were acquired from the Molecular Signatures Database (MSigDB) [25]. The details are listed in Supplementary Table 2. Their activity was evaluated through single-sample gene set enrichment analysis (ssGSEA) [26].

### Inferring immunotherapy response

The gene sets of immune checkpoints (Supplementary Table 3), and immunogenic cell death (ICD) (Supplementary Table 4) were obtained from previous research [27]. The abundance of immune cells was inferred through ssGSEA or ESTIMATE method [28]. Tumor immune dysfunction and exclusion (TIDE) was adopted to estimate immunotherapy response [29]. In addition, this work acquired the transcriptome profiling and clinical outcomes of 348 patients who received anti-PD-L1 antibody atezolizumab in the IMvigor210 cohort. The observed endpoint was the therapeutic response to anti-PD-L1, containing complete response (CR), partial response (PR), stable disease (SD) and progressive disease (PD).

### Definition of a radioresistance scoring (RRscore) system

The DERRGs with *p* < 0.05 were utilized for least absolute shrinkage and selection operator (LASSO) analysis that was run via glmnet approach [30]. Following the minimum lambda, DERRGs (coefficient ≠ 0) were employed for defining RRscore via predict.cv.glmnet function. In accordance with the median RRscore, TCGA-LUAD cases were stratified into low or high RRscore subgroup. OS was compared between subgroups [27], and predictive efficacy was evaluated with receiver operator characteristic curve (ROC). The OS difference between subgroups and the predictive performance of the RRscore were externally proven in the GSE31210 and GSE72094 cohorts. In the GSE30219 cohort, disease-free survival (DFS) was compared between subgroups. TCGA-LUAD patients with radiotherapy were extracted, and survival difference between subgroups was assessed in these populations, followed by evaluation of the efficacy of the RRscore in inferring the survival of patients with radiotherapy. Uni- and multivariate-cox regression analyses were conducted on the RRscore and clinicopathological parameters with LUAD survival. Independent prognostic parameters were retained for constructing a nomogram via rms package [31]. In addition, the predictive accuracy was estimated via calibration curve analysis.

### Patient tissue specimens

Totally, 46 LUAD patients without operative indications who received concurrent chemoradiotherapy were collected from Jiangxi Cancer Hospital. Treatment responses were evaluated through RECIST V.1.1 [32], and 23 patients had PD/SD, and 23 had CR/PR. All tissue samples were immediately frozen in liquid nitrogen. This research followed the Declaration of Helsinki, and gained the approval by the Ethics Committee of Jiangxi Cancer Hospital (2023ky106), with written informed consent obtaining from each patient.

### RNA extraction, RT-PCR and RT-qPCR

Total RNA extraction was conducted utilizing TRIZOL (Sigma-Aldrich, USA), followed by reverse transcription by use of the PrimeScript RT Reagent Kit (Takara, Japan). RT-qPCR was implemented on the ABI Prism 7900 (Applied Biosystems, USA) utilizing SYBR PCR master mix (Takara). Primer sequences comprised (5’-3’): *PTPRH* (GGCGGCACAACAGAGACTC (forward (F)); CTGTGGCAGTAGTGACAGTCC (reverse) (R)), *BEX4* (AAAGAGGAACTAGCGGCAAAC (F); CCAAATGGCGGGATTCTTCTTC (R)), LYPD3 (GATGCTCCCCGAACAAGATGA (F); CAGCGAGAATTGTCCGTGGAT (R)), *FAM83A* (GGCCCTAAGGGACTGGACT (F); CACAGTGGCGCTGGATTTTT (R)), *PLEK2* (GCGATGGTTCATCCTTCGG (F); ATAGCCCCGGTGATCTCAAAG (R)), *ESYT3* (AGACCTGGCCCTACCTAAGC (F); CCTTGACACCGTTGACCCTG (R)), *IFNB1* (ATGACCAACAAGTGTCTCCTCC (F); GGAATCCAAGCAAGTTGTAGCTC (R)), *CCL5* (CCAGCAGTCGTCTTTGTCAC (F); CTCTGGGTTGGCACACACTT (R)), *CXCL10* (GTGGCATTCAAGGAGTACCTC (F); TGATGGCCTTCGATTCTGGATT (R)), and *GAPDH* (GGAGCGAGATCCCTCCAAAAT (F); GGCTGTTGTCATACTTCTCATGG (R)). The relative expression was quantified with 2^−△△Ct^ formula.

### Cell culture, irradiation, and transfection

A549, H358 and LLC cells were purchased from the American Tissue Type Cell Collection (ATCC, USA). A549 and H358 cells were cultivated in RPMI-1640 medium (Gibco, USA) plus 10% fetal bovine serum (Gibco). LLC cells were cultivated in DMEM (Gibco) plus 10% FBS and GLUTAMAX I (Sigma-Aldrich, USA). All the cells were grown in a 5% CO_2_ incubator at 37 °C. To establish radioresistant cells, parent A549 and H358 cells were treated with 18 Gy in three fractions over a period of 7 days, as previously described [33]. Monoclonal cells displaying the highest radioresistance were acquired, namely A549/IR, and H358/IR.

The entire *ESYT3* coding sequence (CDS) sequence was cloned into the pLenti-CMV-puro plasmid (Invitrogen, USA), with empty pLenti-CMV-puro vector as a control (empty vector). Lentivirus was constructed in HEK293 cells with transfection of the pLenti-CMV-puro plasmids, and stable-transfected LUAD cells were finally generated. Small interfering RNAs (siRNAs) targeting *ESYT3* (si-ESYT3#1: 5’-GAGUGAAACAAGGUCAGCAAA-3’; si-ESYT3#2: 5’-UGGUAUGAGCUGACUCCAAAU-3’) were transfected into LUAD cells via Lipofectamine 3000 reagent (Gibco) following the manufacturer’s instructions. To activate STING, cells were treated with 20 nM diABZI STING agonist-1 trihydrochloride (MedChemExpress, USA).

### Cell counting kit-8 (CCK-8)

2 × 10^3^ cells per well were seeded onto a 96-well plate. Following 96 h, 10 µL CCK-8 reagent (Invitrogen) was added to each well. After 1 h, the optical absorbance value at 450 nm was measured.

### Immunofluorescence (IF)

Cells were seeded onto a 24-well culture plate, which were subsequently fixed by methanol at 20 °C for 10 min, and blocked by 1% BSA in PBS for 30 min. Cells were incubated with primary antibody against ESYT3 (1/100; bs-12165R; Bioss, China), γH2AX (1/100; ab229914; Abcam), STING (1/100; ab239074; Abcam) or cGAS (1/50; ab302617; Abcam) at 4 °C overnight, with subsequent incubation with anti-rabbit IgG H&L Alexa Fluor^®^ 488 (1/200; ab150077; Abcam) or CoraLite594 (1/200; SA00013-4; Proteintech, China) for 1 h. DAPI staining was then conducted. Cells were mounted on slides and observed under a confocal microscope (Zeiss, Japan).

### Immunohistochemistry (IHC)

From the Human Protein Atlas (https://www.proteinatlas.org/), IHC staining of ESYT3 antibody (HPA039200) in human LUAD tissues was obtained.

### Colony formation assay

5 × 10^2^ cells were inoculated onto a 6-well plate. Following 14 days, cells were stained by 0.1% crystal violet overnight, with fixation by 4% paraformaldehyde after 20 min. Lastly, colonies > 50 cells were counted.

### Flow cytometry

To evaluate apoptosis, after washing twice by precooled PBS, cell pellets were resuspended in 1× binding buffer. Next, 1 × 10^5^ cells, 5 µL PI and 5 µL Annexin V-FITC (Beyotime, Shanghai, China) were added to binding buffer. After avoiding light for 15 min at room temperature, staining was tested utilizing CytoFLEX flow cytometer (Beckman Coulter, USA). Tumor tissue was processed into single cell suspension after grinding and filtration. CD3^+^CD8^+^ T cells and CD3^−^CD56^+^ NK cells were analyzed. Flow cytometric analysis was performed by use of CytoFLEX flow cytometer.

### Intracellular ROS detection

Cells were incubated with 10 µM DCFH-DA (Sigma, USA) at 37 °C for 20 min. After washing by serum-free culture medium, photographs were acquired utilizing a fluorescence microscope (Zeiss). The fluorescence intensity was measured through ImageJ software.

### RNA sequencing (RNA-seq)

Through Agilent 2100 Bioanalyzer (Agilent Technologies, USA), the extracted RNA was assessed, which was then validated by RNase-free agarose gel electrophoresis. After enrichment of eukaryotic mRNAs and removing rRNAs, the enriched mRNA was segmented into short fragments and reverse transcribed into cDNA. RNA-seq was conducted through Illumina NovaSeq6000. DEGs were identified with |log2FC|>1 & adjusted *p* < 0.05, and functional enrichment analysis was performed.

### Coimmunoprecipitation (Co-IP) and coimmunoprecipitation-based mass spectrometry (Co-IP-MS)

Protein A/G magnetic beads (Thermo Fisher, USA) were utilized to carried out Co-IP. Cells were washed with 1× PBS for three times, which were subsequently lysed on ice in IP lysis buffer with protease inhibitors (cocktail) for 1 h. Total protein was separated utilizing SDS–PAGE, and transferred onto PVDF membranes. The protein pre-cleared by magnetic beads was incubated with primary antibody against ESYT3 (bs-12165R; Bioss) or STING (ab239074; Abcam) overnight at 4 °C. Following adsorbing magnetic beads, the protein was denatured. Western blot was eventually implemented. For Co-IP-MS, protein samples were subjected to western blot or SDS-PAGE, with subsequent Coomassie staining. Gel pieces were cut off and mass spectrometry was performed.

### Western blot

Proteins were separated via SDS–PAGE (6% or 10%) and transferred onto PVDF membranes. The membranes were blocked with 5% milk/TBST for 1 h at room temperature, and incubated with primary antibody against ESYT3 (1/1000; bs-12165R; Bioss), STING (1/20000; 66680-1-Ig; Proteintech), p-STING (1/1000; ab318181; Abcam), cGAS (1/2000; 26416-1-AP; Proteintech), IgG (1/20000; 30000-0-AP; Proteintech), p-TBK1 (1/5000; ab109272; Abcam), p-IRF3 (1/2000; 29528-1-AP; Proteintech) or GAPDH (1/50000; 60004-1-Ig; Proteintech) at 4 °C overnight. Subsequently, the membranes were incubated with HRP conjugated goat anti-rabbit or anti-mouse secondary antibody (1/10000; A21020 or A21010; abbkine, China). Bands were visualized by use of enhanced chemiluminescence (ECL) kit (GE Healthcare, USA). Images were captured through automatic ECL imaging system (Bio-Rad, USA).

### Enzyme-linked immunosorbent assay (ELISA)

The levels of IFNβ, CCL5 and CXCL10 in culture supernatant were detected utilizing commercial ELISA assay kits of IFNβ, CCL5 and CXCL10 following manufacturer’s instructions (H024-1-1, H496-1 and H495-1, respectively; Nanjing Jiancheng Bioengineering Institute, China).

### Mitochondrial DNA (mtDNA) detection

Genomic DNA (gDNA) extraction was achieved through boiling approach, with subsequent cytoplasmic DNA extraction through digestion approach. Cells were digested via trypsin, washed with PBS twice, and added with 100 µL of 50 µM NaOH. Afterwards, cells were boiled at 98 °C for 15 min, added with 10 µL of 1 M Tris-HCl (PH = 8.0), swirled for 10 s and stood at 4 °C for 30 s. Subsequently, the supernatant was removed. Incubation with 10 µL of 25 mg/mL Protein K at 60 °C was performed for 45 min. After purifying and concentrating gDNA, cells were incubated with 200 µL cytosolic DNA extract buffer on ice for 15 min. They were centrifuged (13,000× g) at 4 °C for 2 min, with subsequent removal of the supernatant. Cells were treated with 10 µL of 25 mg/mL Protein K at 60 °C for 45 min. Subsequently, cytosolic DNA was collected using DNA concentrator kit (ab156895; Abcam), and quantified via qPCR.

### Animal experiment

Female BALB/c nude mice and female C57BL/6 mice were acquired from the Vital River Laboratories Animal Technology (Beijing, China). To construct the xenograft tumor model, ESYT3-overexpressing (OE-ESYT3) or control luciferase-tagged A549 cells (5 × 10^6^ per mouse) were subcutaneously injected into the armpits of BALB/c nude mice (4–5 weeks). After 7 days, tumor volume was monitored every three days. When the tumor reached ~ 50 mm^3^, the mice were irradiated with 5 Gy twice every 6 days. After 35 days, they were euthanized, and tumors were dissected and weighed. Bioluminescence signals were detected utilizing the IVIS Spectrum system (PerkinElmer, USA). To establish the syngeneic mouse model, LLC cells transfected with empty vector or OE-ESYT3 plasmid vector (5 × 10^6^ per mouse) were subcutaneously injected into the armpits of female C57BL/6 mice (4–5 weeks). When the tumor reached ~ 50 mm^3^, the C57BL/6 mice received 5 Gy irradiation treatment twice every 6 days. After 35 days, the mice were euthanized and tumors were gathered. All animal procedures were approved by the Institutional Animal Care and Use Committee of YuanDong International Academy of Life Sciences.

### Statistical analysis

All the analyses were conducted utilizing R packages (version 3.6.1) or GraphPad Prism software (version 9.0.1). Variables between groups was compared via student’s t, Wilcoxon, or one- or two-way ANOVA test. Kaplan-Meier survival curves were drawn through survival and survminer packages, and were analyzed through log-rank test. Pearson’s or Spearman’s test was adopted for correlation analysis. *P* < 0.05 was indicative of statistical difference.

## Results

### Development of a radiotherapeutic response classification for LUAD

In total, 3117 genes displayed aberrant expression in LUAD versus controls (Supplementary Fig. 1A, B; Supplementary Table 5). After intersecting with RRGs, we determined 608 DERRGs (Supplementary Fig. 1C), among which 190 were significantly linked with LUAD prognosis (Supplementary Table 6) that were potentially connected with radioresistance. Based upon the transcript levels of the prognostic DERRGs, TCGA-LUAD patients were classified as radioresistant subtype (C1) and radiosensitive subtype (C2) (Supplementary Fig. 1D). C2 subtype presented better OS versus C1 (Supplementary Fig. 1E). Most RRGs that were up-regulated in radioresistant LUAD cells presented the remarkable overexpression in C1, while those that were down-regulated in radioresistant LUAD cells exhibited the prominent overexpression in C2 (Supplementary Fig. 1F), further proving C1 as radioresistant subtype and C2 as radiosensitive subtype. The difference in stage was found between subtypes, with more cases with advanced stage in C1 (Supplementary Fig. 1G). Furthermore, the two subtypes were notably different at the transcript levels (Supplementary Fig. 1H). It was also investigated that ROS level was higher in C1 versus C2 subtype (Supplementary Fig. 1I). As illustrated in Supplementary Fig. 1J, C2 presented the higher responses to UV and X-ray, indicating that C2 patients were more sensitive to radiotherapy. In addition, most DNA damage repair pathways had the higher levels in C1 subtype (Supplementary Fig. 1K), indicating the radioresistance in this subtype.

Ionizing radiation triggers ferroptosis in tumor cells, and ferroptosis activation can sensitize radioresistant tumor cells [34]. In comparison to C2 subtype, most ferroptosis inducers presented the lower expression in C1, with the higher expression of most suppressors (Supplementary Fig. 1L), indicating the ferroptosis inhibition in radioresistant patients. In addition, more ICD genes presented the higher level in C2 versus C1 subtype (Supplementary Fig. 1M). The infiltration levels of most immune cells were lower in C1 subtype (Supplementary Fig. 1N). Furthermore, C1 subtype had the lower immune/stromal score, and higher tumor purity (Supplementary Fig. 1O-Q). These data proved the immunosuppression in the radioresistant subtype. We also adopted TIDE to infer immunotherapy response. Consequently, C2 subtype was more likely to respond to immunotherapy based upon the higher dysfunction score and the lower exclusion score and TIDE score (Supplementary Fig. 1R-T).

#### Genetic mutation heterogeneity between radioresistant and radiosensitive subtypes

The widespread somatic mutations and copy-number amplification and deletion might contribute to the aberrant expression of DERRGs (Supplementary Fig. 2A-C). More frequent copy-number amplifications and deletions were observed in C1 relative to C2 subtype (Supplementary Fig. 1A-D). In addition, C1 presented the higher frequency of somatic mutations in comparison to C2 (Supplementary Fig. 3E, F). Overall, higher TMB score was demonstrated in C1 versus C2 subtype (Supplementary Fig. 1G). These findings proved the heterogeneity in genetic mutations between the radioresistant and radiosensitive subtypes.

#### Assessment of the reliability and repeatability of the radiotherapeutic response classification

Two LUAD cohorts: GSE31210 and GSE72094, were utilized for further verifying the radioresistant and radiosensitive subtypes. This work determined the top 100 up-regulated marker genes of each subtype in the GSE31210 cohort, respectively (Supplementary Fig. 4A). After quantifying and evaluating prediction confidence, samples with *p* < 0.05 were extracted for further observation (Supplementary Fig. 4B). It was proven that C2 possessed the longer OS in the GSE31210 cohort (Supplementary Fig. 4D-F). Above data demonstrated the stability and repeatability of the classification.

### Quantification of the radiotherapeutic response classification for clinical application

The prognostic DERRGs were utilized for feature selection via LASSO analysis. Under the minimum lambda value (Supplementary Fig. 5A, B), the DERRGs with coefficient ≠ 0 were retained for generating a RRscore system following the formula: RRscore = 0.0920 * *FAM83A* expression + 0.0776 * *PLEK2* expression + 0.0247 * *PTPRH* expression + (-0.0668) * *BEX4* expression + (-0.0349) * *ESYT3* expression + 0.0668 * *LYPD3* (Supplementary Fig. 5C). The AUC values at 1-, 3- and 5-year OS were all > 0.60, demonstrating that the RRscore was accurately predictive of patient survival (Supplementary Fig. 5D). High RRscore individuals owned the shorter OS time in comparison to those with low RRscore (Supplementary Fig. 5E). From the uni- and multivariate-cox regression results, the RRscore was an independent risk factor, except for stage (Supplementary Fig. 5F, G). For the promotion of the RRscore in clinical practice, we proposed a nomogram composed of the RRscore and stage (Supplementary Fig. 5H). It was demonstrated that the nomogram owned the excellent efficacy in LUAD prognostication (Supplementary Fig. 5I).

It was found that the RRscore presented the notably positive interactions with X-ray and UV responses and ROS (Supplementary Fig. 5J). Additionally, the RRscore presented positive interactions with most DNA damage repair processes, such as base excision repair, editing and processing nucleases, Fanconi anemia, homologous recombination, non-homologous end-joining, repair of DNA-protein crosslinks (Supplementary Fig. 5K). The RRscore presented the strongly positive connections with *CD274* (PD-L1) and *CD276* (B7H3) (Supplementary Fig. 5L). In addition, there were notable interactions of the RRscore with ICD molecules (*PANX1*,* PPIA*, etc.) in LUAD (Supplementary Fig. 5M). As illustrated in Supplementary Fig. 5N, the RRscore was strongly and positively connected with activated CD4^+^ T cells and neutrophils. In the IMvigor210 cohort, the higher percentage of patients who responded to anti-PD-L1 therapy was found in the low RRscore group (Supplementary Fig. 5O). Moreover, patients with low RRscore owned the better OS outcomes versus those with high RRscore (Supplementary Fig. 5P). It was also observed the lower RRscore in responders versus non-responders (Supplementary Fig. 5Q). The response to immunotherapy was inferred by use of TIDE algorithm. Based upon the higher dysfunction score, lower exclusion score and TIDE score, patients with low RRscore more possibly benefited from immunotherapy (Supplementary Fig. 5R-T).

To prove the reliability of the RRscore in prognostication, multiple cohorts were adopted for external validation. Both in the GSE31210 (Supplementary Fig. 6A-C) and GSE72094 (Supplementary Fig. 6D-F) cohorts, shorter OS was demonstrated in high-RRscore patients, and AUC values at one-, three-, or five-year OS all exceeded 0.60. It was also shown that high-RRscore patients exhibited poorer DFS in the GSE30219 cohort (Supplementary Fig. 6G). This work further evaluated whether the RRscore can infer survival outcomes of patients who received radiotherapy. Consequently, worse OS outcomes were proven in high-risk individuals, with AUC values of one-, three-, or five-year survival > 0.7 (Supplementary Fig. 6H, I), suggesting that the RRscore might be used for predicting the benefit from radiotherapy.

### ESYT3 expression is attenuated in the context of LUAD radioresistance

**Fig. 1:**
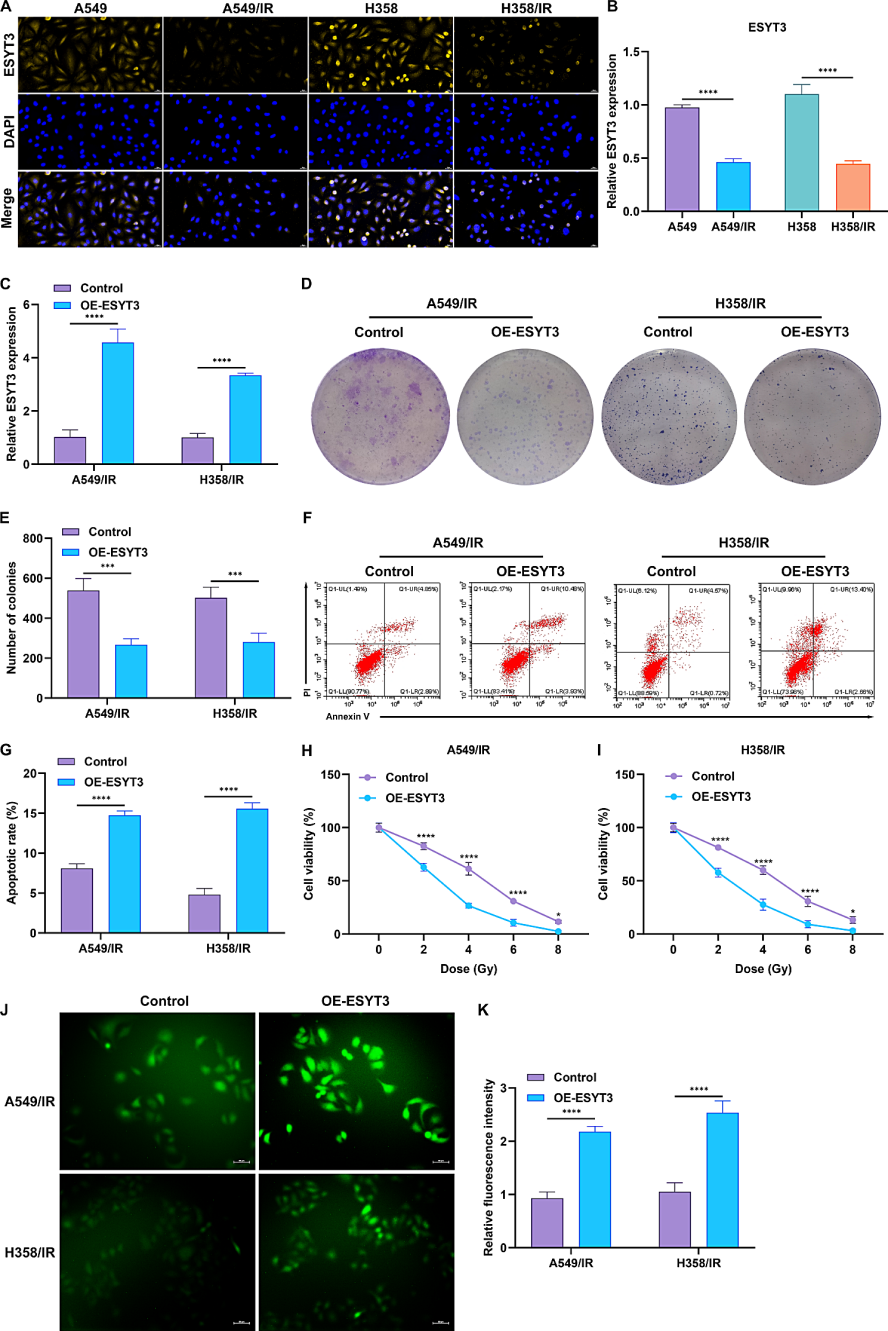
Overexpression of ESYT3 attenuates LUAD radioresistance. (**A**, **B**) IF staining analysis of ESYT3 expression in radiosensitive and radioresistant LUAD cells. Bar, 20 μm. (**C**) RT-qPCR analysis of ESYT3 expression in radioresistant LUAD cells transfected with ESYT3 overexpression or empty vector. (**D**, **E**) Colony formation of radioresistant LUAD cells with control or ESYT3 overexpression. (**F**, **G**) Apoptotic levels of radioresistant LUAD cells with control or ESYT3 overexpression. (**H**, **I**) Cell survival of radioresistant LUAD cells with control or ESYT3 overexpression upon distinct dosages of irradiation. (**J**, **K**) IF staining of DCFH-DA-labeled ROS in radioresistant LUAD cells with control or ESYT3 overexpression as well as quantification of the fluorescence intensity. Bar, 50 μm. *n* = 3 for each experiment. *p < 0.05; ***p < 0.001; ****p < 0.0001

This study gathered 46 LUAD patients who had no operative indications and received concurrent chemoradiotherapy, including 23 cases with PD/SD, and 23 with CR/PR. The expression of the prognostic DERRGs in the RRscore system was then measured. PTPRH, BEX4, and LYPD3 presented higher expression in PD/SD versus CR/PR, with lower expression of FAM83A, PLEK2, and ESYT3 (Supplementary Fig. 7A-F), reflecting the relationships between the DERRGs and radiotherapeutic response. For mimicking the residual cells following radiotherapy, radioresistant cells (A549/IR and H1975/IR) were constructed following exposure of A549 and H1975 cells to 18 Gy irradiation in three fractions. The two radioresistant cells displayed stronger survival capacities under irradiation in comparison to their parental cells (Supplementary Fig. 7G, H). The transcript levels of the prognostic DERRGs in the RRscore system were further verified in radiosensitive and radioresistant LUAD cells. As expected, PTPRH, BEX4, and LYPD3 exhibited the remarkable overexpression in the two radioresistant cells than corresponding parental cells, with the down-regulation of FAM83A, PLEK2, and ESYT3 (Supplementary Fig. 7I-P). Among the prognostic DERRGs from the RRscore system, ESYT3 expression was down-regulated in LUAD than normal tissues, and was negatively associated with pathological stage (Supplementary Fig. 8A, B), which was consistent with a prior bioinformatics study [35]. Additionally, high ESYT3 expression was connected to prolonged OS and DFS of LUAD patients (Supplementary Fig. 8C, D). Especially, ESYT3 was nearly not expressed in human LUAD tissue samples (Supplementary Fig. 8E). It was also proven that ESYT3 expression was reduced in the two radioresistant cells versus corresponding parental cells (Fig. 1A, B). Therefore, ESYT3 aroused our great interest, and we infer that ESYT3 down-regulation is related to LUAD radioresistance.

## Overexpression of ESYT3 alleviates radioresistance of LUAD cells

To evaluate whether ESYT3 influenced radiotherapeutic response in LUAD, we overexpressed ESYT3 in A549/IR and H1975/IR cells through transfecting ESYT3 overexpression plasmid (Fig. 1C; Supplementary Fig. 9A). Consequently, overexpressed ESYT3 mitigated the proliferative ability of radioresistant LUAD cells (Fig. 1D, E). In addition, apoptotic level in radioresistant LUAD cells was remarkably increased by ESYT3 overexpression (Fig. 1F, G). Next, this study assessed the influence of ESYT3 on DNA damage for irradiation at distinct time points. Consequently, the proliferation following irradiation in A549/IR and H1975/IR cells overexpressing ESYT3 was lower, indicating that LUAD cells with overexpressed ESYT3 was more sensitive to DNA damage response (Fig. 1H, I). Interestingly, intracellular ROS accumulation was also induced by ESYT3 overexpression in two radioresistant LUAD cells (Fig. 1J, K). Based upon above evidence, this study infers that ESYT3 overexpression enables to attenuate LUAD radioresistance, which is related to DNA damage mechanisms.

### Overexpression of ESYT3 sensitives LUAD cells to DNA damage induced by irradiation

**Fig. 2:**
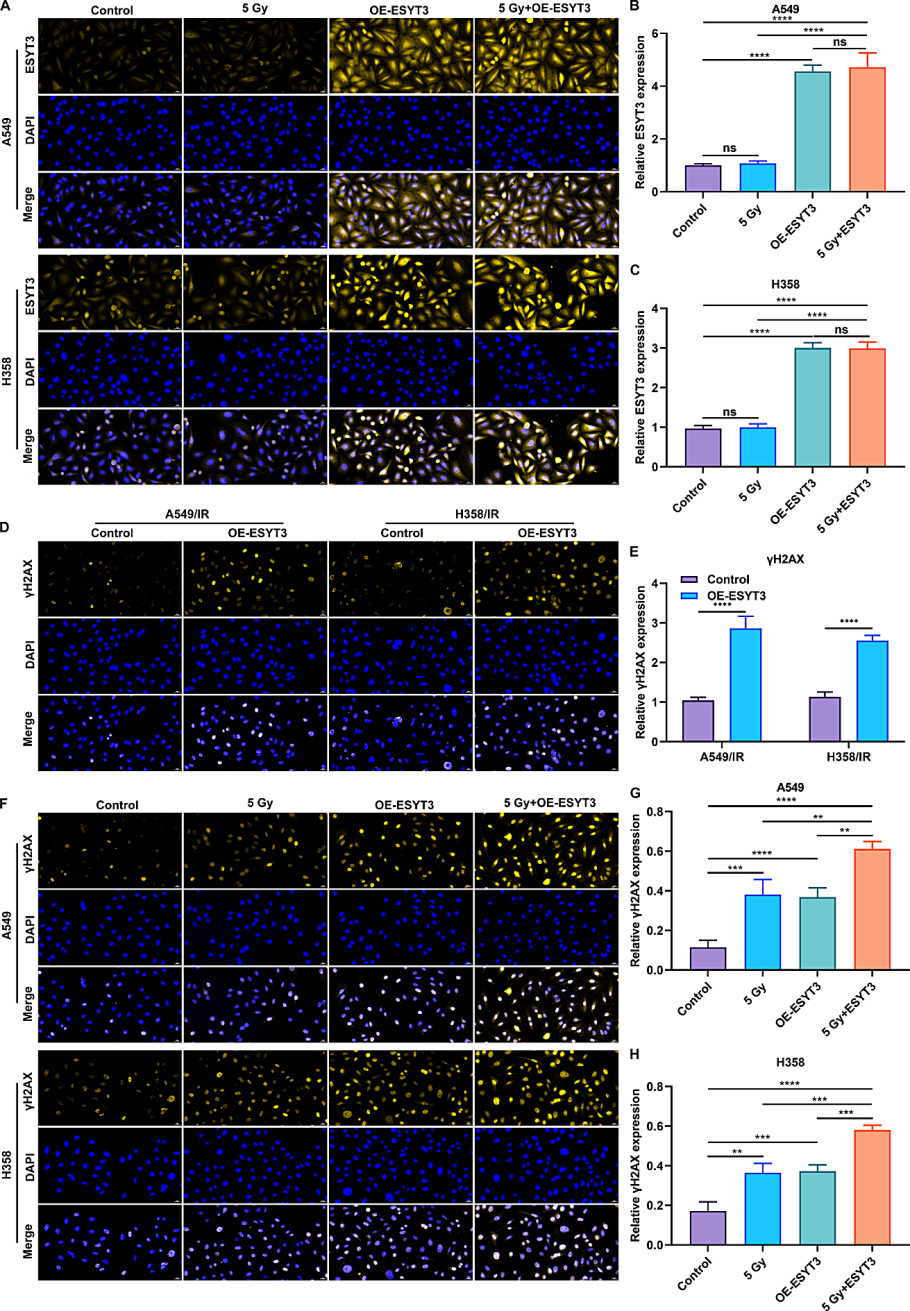
Overexpression of ESYT3 sensitives LUAD cells to DNA damage induced by irradiation. (**A**-**C**) IF analysis of ESYT3 expression in A549 and H1975 cells with ESYT3 overexpression for 2 h following irradiation treatment. Bar, 20 μm. (**D**, **E**) IF analysis of γH2AX expression in A549/IR and H1975/IR cells with ESYT3 overexpression. Bar, 20 μm. (**F**-**H**) IF analysis of γH2AX expression in A549 and H1975 cells with ESYT3 overexpression for 2 h following irradiation. Bar, 20 μm. *n* = 3 for each experiment. **p < 0.01; ***p < 0.001; ****p < 0.0001; ns: *p* > 0.05

Although ESYT3 expression was reduced in LUAD radioresistance, we found that irradiation cannot influence the expression of ESYT3 in LUAD cells (Fig. 2A-C). DNA damage was assessed through staining the cells with γH2AX antibody. Intriguingly, the fluorescence intensity of γH2AX was elevated in radioresistant (Fig. 2D, E) or radiosensitive (Fig. 2F-H) LUAD cells overexpressing ESYT3, implying that overexpression of ESYT3 can induce DNA damage of LUAD cells regardless of radioresistance. More importantly, LUAD cells overexpressing ESYT3 displayed the higher fluorescence intensity of γH2AX upon irradiation (Fig. 2F-H). Based upon these findings, overexpression of ESYT3 can sensitive LUAD cells to irradiation-induced DNA damage response.

### ESYT3 interacts with STING in LUAD

**Fig. 3:**
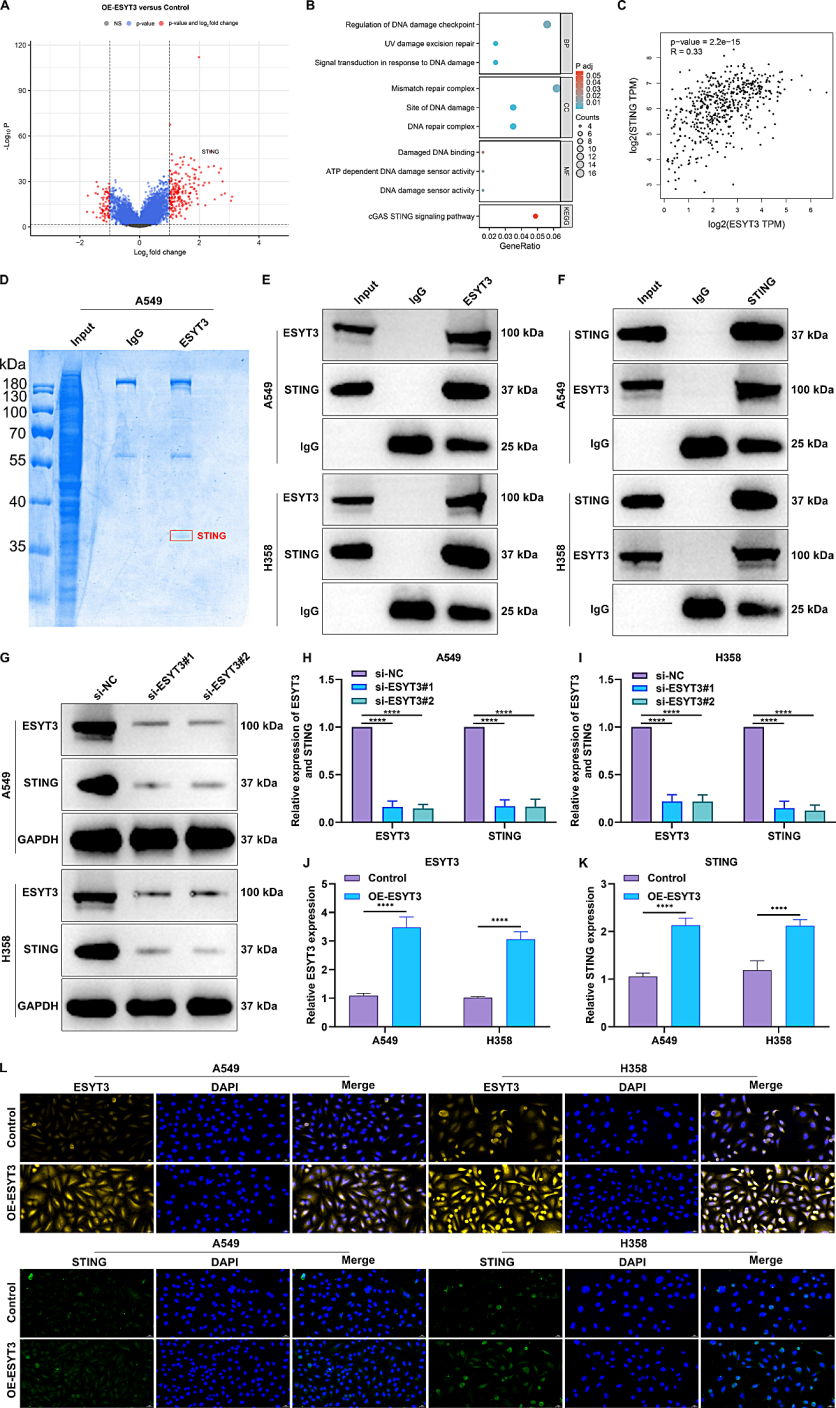
ESYT3 interacts with STING in LUAD. (**A**) RNA-seq analysis of DEGs between control and ESYT3-overexpressed A549 cells. (**B**) Functional enrichment analysis of the DEGs. (**C**) Scatter plots displaying the association between ESYT3 and STING across TCGA-LUAD samples. (**D**) Co-IP-MS for exploring the interactive proteins of ESYT3. (**E**, **F**) Co-IP analysis of the interaction between ESYT3 and STING in A549 and H1975 cells. (**G**-**I**) Western blot for detecting ESYT3 and STING expression in A549 and H1975 cells after transfection of ESYT3 siRNAs. (**J**-**L**) IF staining of ESYT3 and STING expression in the two LUAD cells after overexpressing ESYT3. Bar, 20 μm. *n* = 3 for each experiment. ****p < 0.0001

To explore the molecular mechanisms of ESYT3, we performed RNA-seq analysis on control and ESYT3-overexpressed LUAD cells. The results showed that there were 81 down-regulated genes and 219 up-regulated genes in OE-ESYT3 LUAD cells compared to controls (Fig. 3A). Furthermore, we found that the DEGs were significantly linked to DNA damage-related processes and pathways, especially cGAS-STING signaling pathway (Fig. 3B). RNA-seq analysis showed that STING expression was significantly up-regulated in OE-ESYT3 LUAD cells compared to controls (Fig. 3A). Through the website (https://www.genecards.org/), ESYT3 was predicted to be abundant in endoplasmic reticulum and plasma membrane, but less abundant in the cytosol and nucleus. Meanwhile, STING acts as an endoplasmic reticulum transmembrane protein, which is primarily expressed in endoplasmic reticulum membrane, plasma membrane, nuclei, and mitochondria. Therefore, we hypothesize that ESYT3 was co-localized with STING. A positive association between ESYT3 and STING was found in TCGA-LUAD samples (Fig. 3C). To further elucidate the functional mechanism of ESYT3 in LUAD, Co-IP-MS was performed to investigate interactive proteins of ESYT3. STING was identified as one of the interactive proteins of ESYT3 (Fig. 3D). Co-IP also confirmed the direct interaction between ESYT3 and STING in two LUAD cells (Fig. 3E, F). In addition, it was found that ESYT3 knockdown significantly decreased STING expression (Fig. 3G-I). In contrast, overexpression of ESYT3 significantly increased STING expression (Fig. 3J-L). The interaction between ESYT3 and STING was also proven by molecular docking (Supplementary Fig. 10A, B). Altogether, above findings uncover that ESYT3 interacts with STING in LUAD.

#### ESYT3 activates cGAS-STING-dependent DNA damage signaling in LUAD

**Fig. 4:**
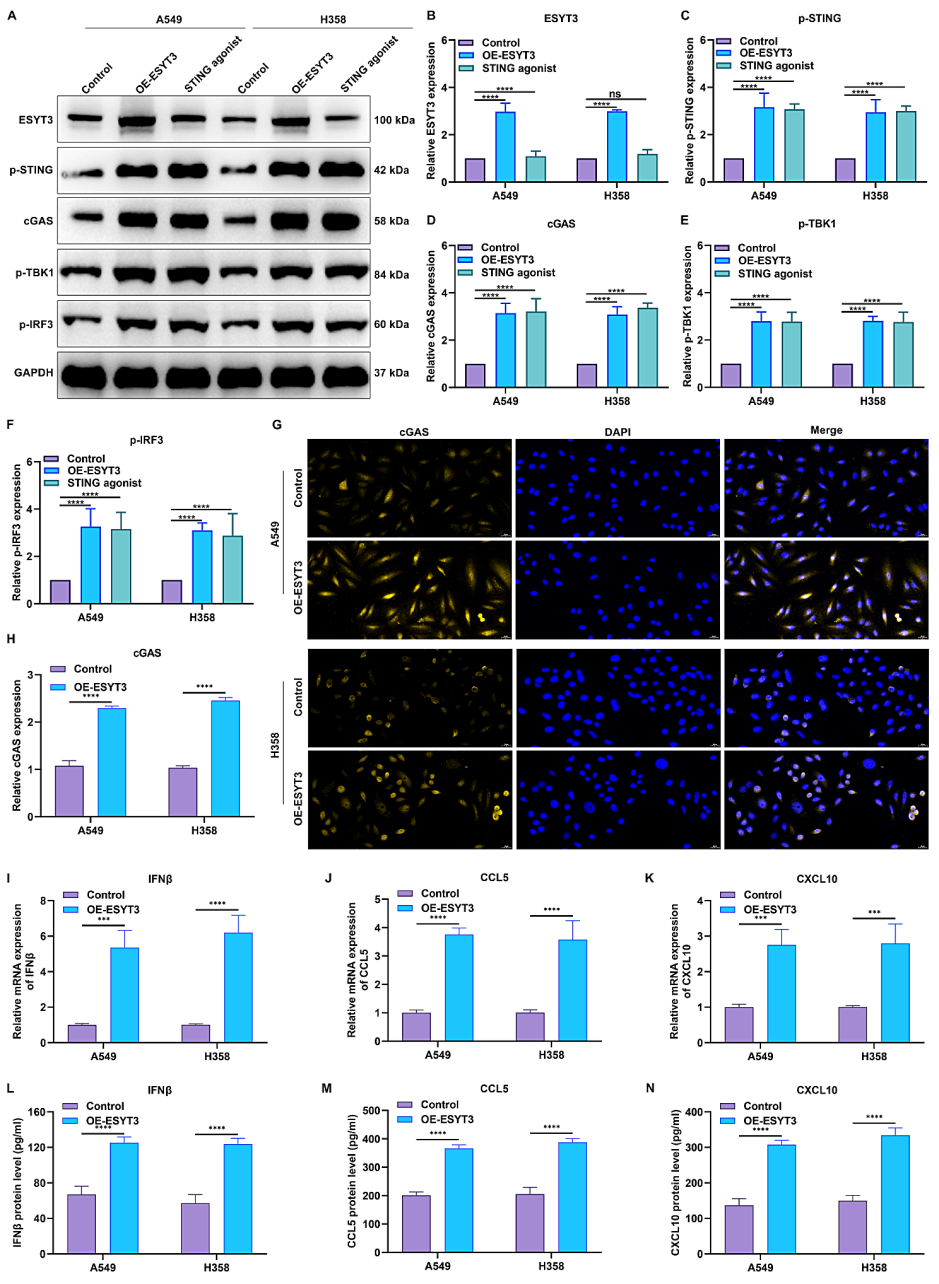
ESYT3 activates type I IFN responses via cGAS-STING signaling in LUAD. (**A**-**F**) Western blot for detecting ESYT3, p-STING, cGAS, p-TBK1 and p-IRF3 in A549 and H1975 cells transfected with ESYT3 overexpression plasmids or treated with STING agonist. (**G**, **H**) IF staining of cGAS expression in two LUAD cells overexpressing ESYT3. Bar, 20 μm. (**I**-**K**) RT-qPCR for the mRNA expression levels of IFNβ, CCL5 and CXCL10 in two LUAD cells with ESYT3 overexpression. (**L**-**N**) ELISA of IFNβ, CCL5 and CXCL10 levels in the supernatant of ESYT3-overexpressing LUAD cells. *n* = 3 for each experiment. ***p < 0.001; ****p < 0.0001; ns: *p* > 0.05

Overexpression of ESYT3 significantly increased the levels of ESYT3, p-STING, cGAS, p-TBK1 and p-IRF3 in A549 and H1975 cells, and the results were similar to STING agonist treatment, demonstrating that ESYT3 was involved in mediating cGAS-STING signaling pathway (Fig. 4A-F). IF analysis also confirmed that ESYT3 overexpression induced the remarkable up-regulation of cGAS in LUAD cells (Fig. 4G, H). Furthermore, ESYT3 overexpression enhanced the mRNA expression and production of IFNβ, CCL5 and CXCL10 (Supplementary Fig. 9A; Fig. 4I-N). These findings uncover that ESYT3 activates cGAS-STING-dependent DNA damage signaling in LUAD, and subsequently increases the generation of type I IFNs and downstream chemokines CCL5 and CXCL10, eventually improving radioimmune responses.

#### ESYT3 improves radioimmune responses of LUAD cells through activating cGAS-STING-dependent signaling

**Fig. 5:**
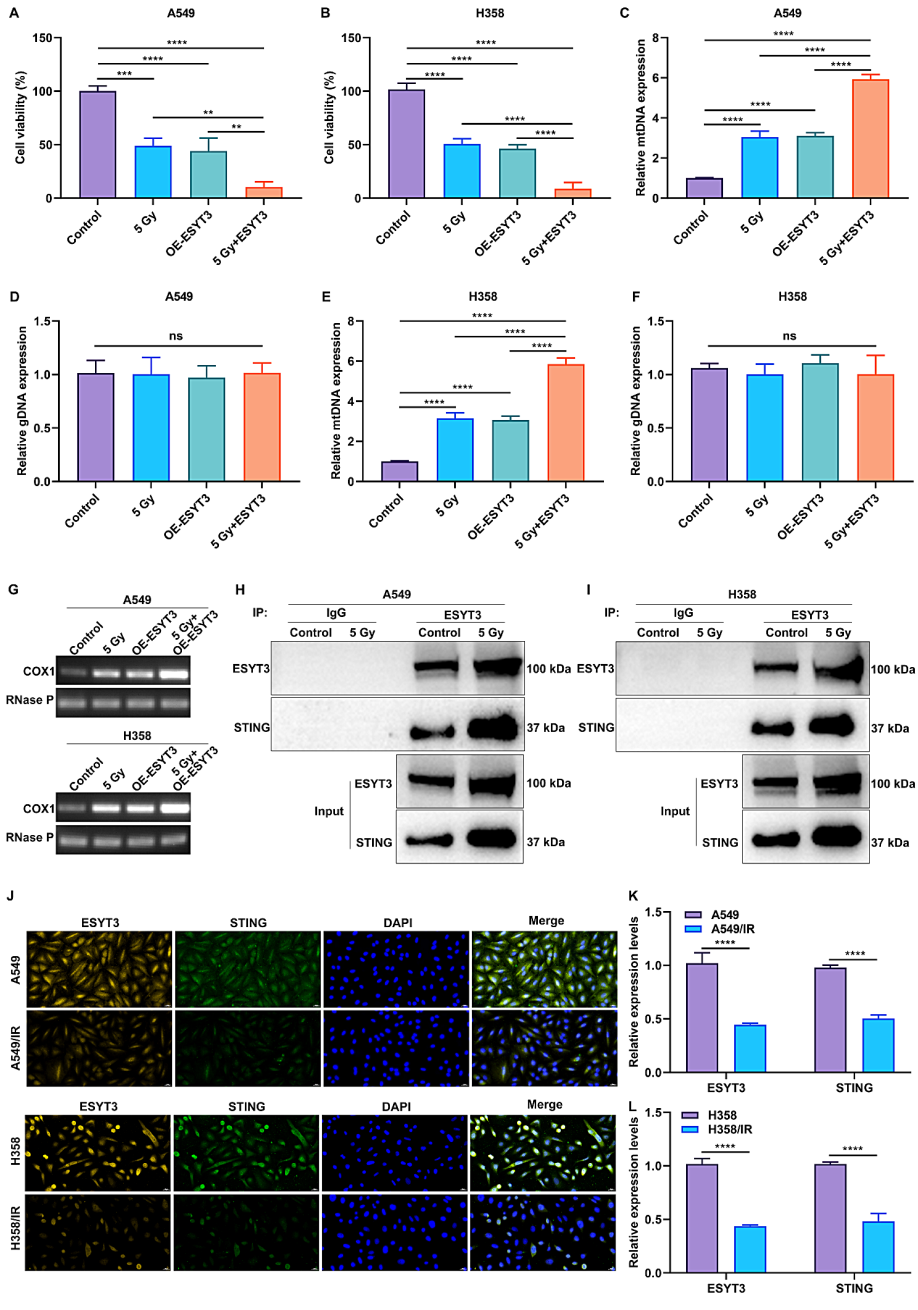
ESYT3 facilitates DNA damage response in STING-dependent signaling. (**A**, **B**) CCK-8 for the proliferation of A549 and H1975 cells with ESYT3 overexpression for 2 h following irradiation induction. (**C**-**G**) Analysis of mtDNA in two LUAD cells with or without irradiation induction for 6 h after overexpressing ESYT3. (**H**, **I**) Co-IP analysis of the interaction between ESYT3 and STING in two LUAD cells upon irradiation (5 Gy) for 2 h. (**J**-**L**) Multiple IF staining for detecting the subcellular co-localization of ESYT3 and STING both in radiosensitive and radioresistant LUAD cells. Bar, 20 μm. *n* = 3 for each experiment. **p < 0.01; ***p < 0.001; ****p < 0.0001; ns: *p* > 0.05

**Fig. 6:**
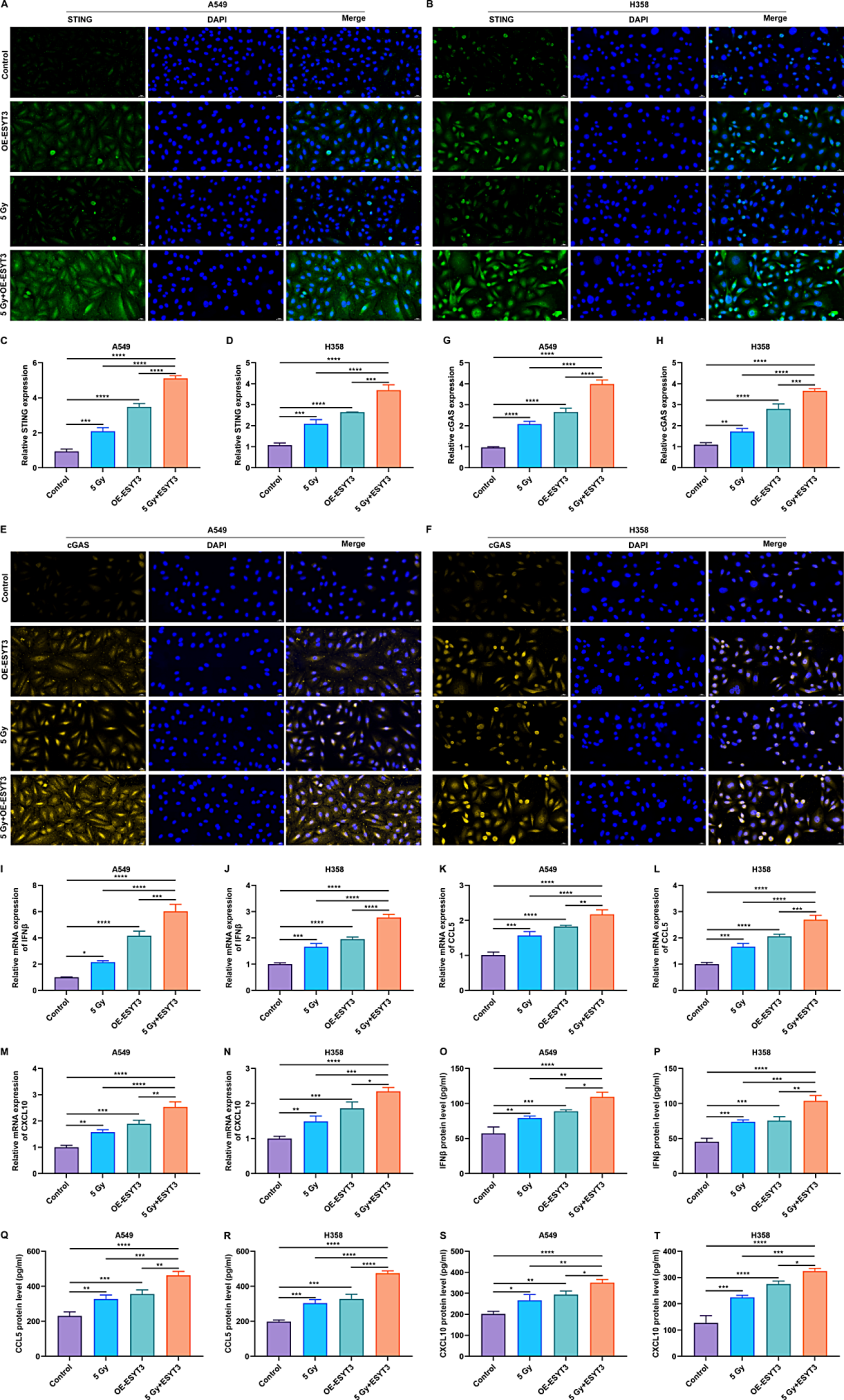
ESYT3 improves radioimmune responses of LUAD cells through activating cGAS-STING-dependent signaling. (**A**-**D**) IF staining of STING expression in A549 and H1975 cells overexpressing ESYT3 following irradiation induction (5 Gy) for 2 h. Bar, 20 μm. (**E**-**H**) IF staining detecting cGAS expression in two LUAD cells overexpressing ESYT3 following irradiation induction (5 Gy) for 2 h. (**I**-**N**) RT-qPCR analysis of the transcript levels of IFNβ, CCL5 and CXCL10. (**O**-**T**) ELISA of the production of IFNβ, CCL5 and CXCL10. Bar, 20 μm. *n* = 3 for each experiment. *p < 0.05; **p < 0.01; ***p < 0.001; ****p < 0.0001

As expected, overexpression of ESYT3 cooperated with irradiation to synergistically inhibit proliferation of LUAD cells (Fig. 5A, B), implying the enhanced sensitivity of LUAD cells to DNA damage response. To identify whether the DNA damage response underlying ESYT3 occurred via activating the cGAS-STING signaling, this study evaluated the altered cytoplasmic DNA distribution in LUAD cells at the molecular level. Following irradiation, mtDNA level was notably higher in LUAD cells with overexpressed ESYT3, without changes in gDNA level (Fig. 5C-G). This implies that overexpressed ESYT3 can contribute to the release of DNA from the nucleus to the cytoplasm, while irradiation facilitates this process. Co-IP results proved that the direct interaction between ESYT3 and STING was remarkably enhanced upon irradiation induction (Fig. 5H, I). Furthermore, IF staining further demonstrated the subcellular co-localization of ESYT3 and STING in LUAD cells (Fig. 5J-L). Moreover, STING and cGAS were up-regulated in LUAD cells after overexpressing ESYT3 or irradiation, while the upregulated levels were much more significant upon ESYT3 overexpression plus irradiation (Fig. 6A-H). In LUAD cells, ESYT3 overexpression and irradiation synergistically elevated the transcription and production of IFNβ, CCL5 and CXCL10 (Supplementary Fig. 9B, C; Fig. 6I-T). Thus, overexpressed ESYT3 improves radioimmune responses of LUAD cells via activating cGAS-STING-dependent signaling.

#### ESYT3 overexpression synergizes with radiotherapy to suppress in vivo tumor growth

**Fig. 7:**
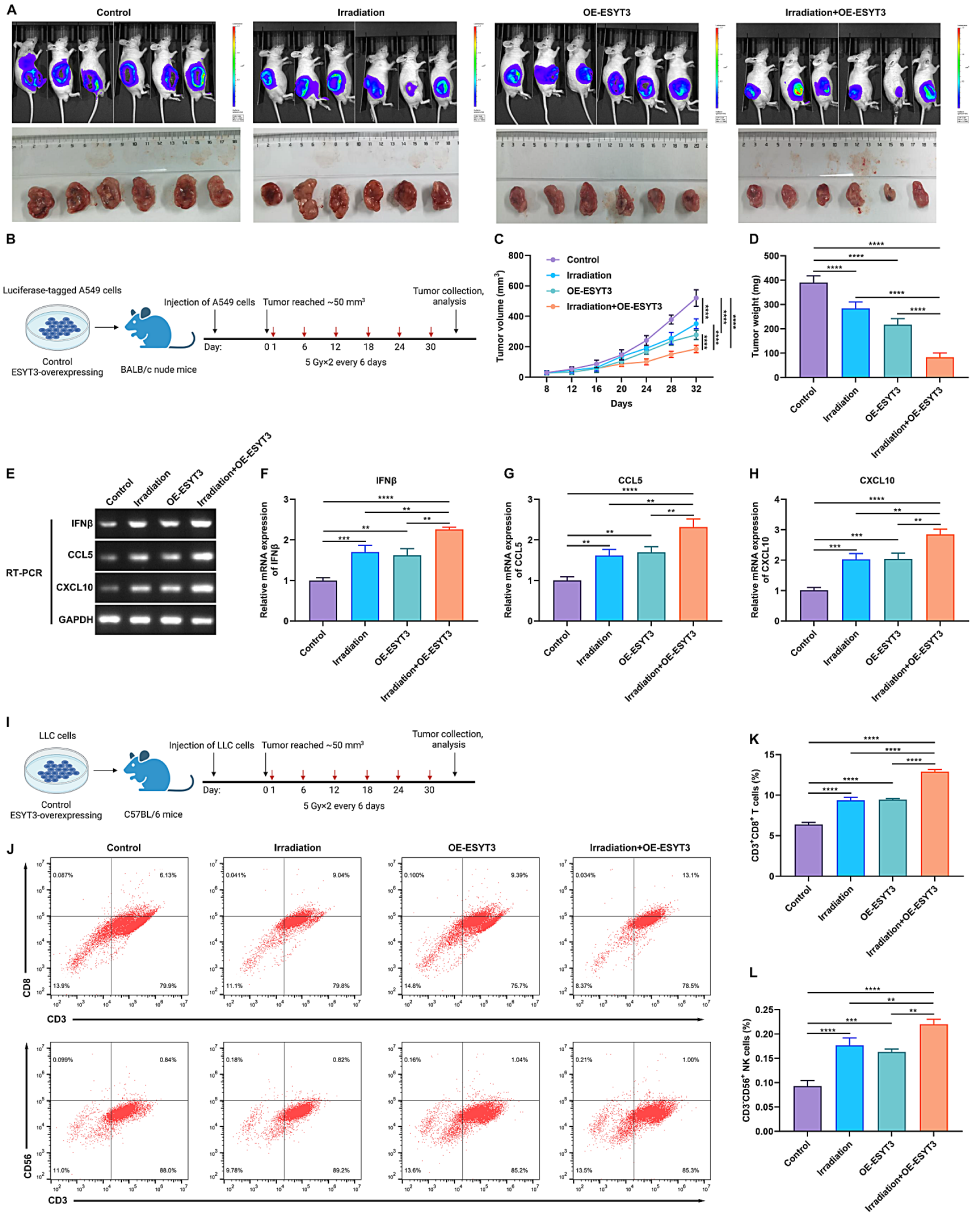
ESYT3 overexpression synergizes with radiotherapy to suppress tumor growth and improve antitumor immune responses. (**A**) Representative images of BALB/c nude mice at day 32 following inoculation of ESYT3-overexpressing (OE-ESYT3) or control luciferase-tagged A549 cells. (**B**) Schematic diagram displaying the grouping and treatment plan of the nude mouse model. BALB/c nude mice were inoculated OE-ESYT3 or control luciferase-tagged A549 cells. When the tumor reached ~ 50 mm^3^, the mice in the irradiation group or irradiation + OE-ESYT3 group were irradiated with 5 Gy twice every 6 days. *n* = 6 each group. (**C**, **D**) Tumor growth curve and tumor weight. (**E**) Representative RT-PCR images of IFNβ, CCL5 and CXCL10 in tumors. (**F**-**H**) Quantification of the expression of IFNβ, CCL5 and CXCL10 in tumors. (**I**) Schematic diagram displaying the grouping and treatment plan of the syngeneic mouse model. C57BL/6 mice were inoculated with inoculated OE-ESYT3 or control LLC cells, and were irradiated at the indicated time points. *n* = 6 each group. (**J**-**L**) Representative images and quantitation of flow cytometry analysis of the percentage of CD3^+^CD8^+^ T cells and CD3^−^CD56^+^ NK cells in tumors from the syngeneic mouse model. There are 6 mice in each group. **p < 0.01; ***p < 0.001; ****p < 0.0001

**Fig. 8:**
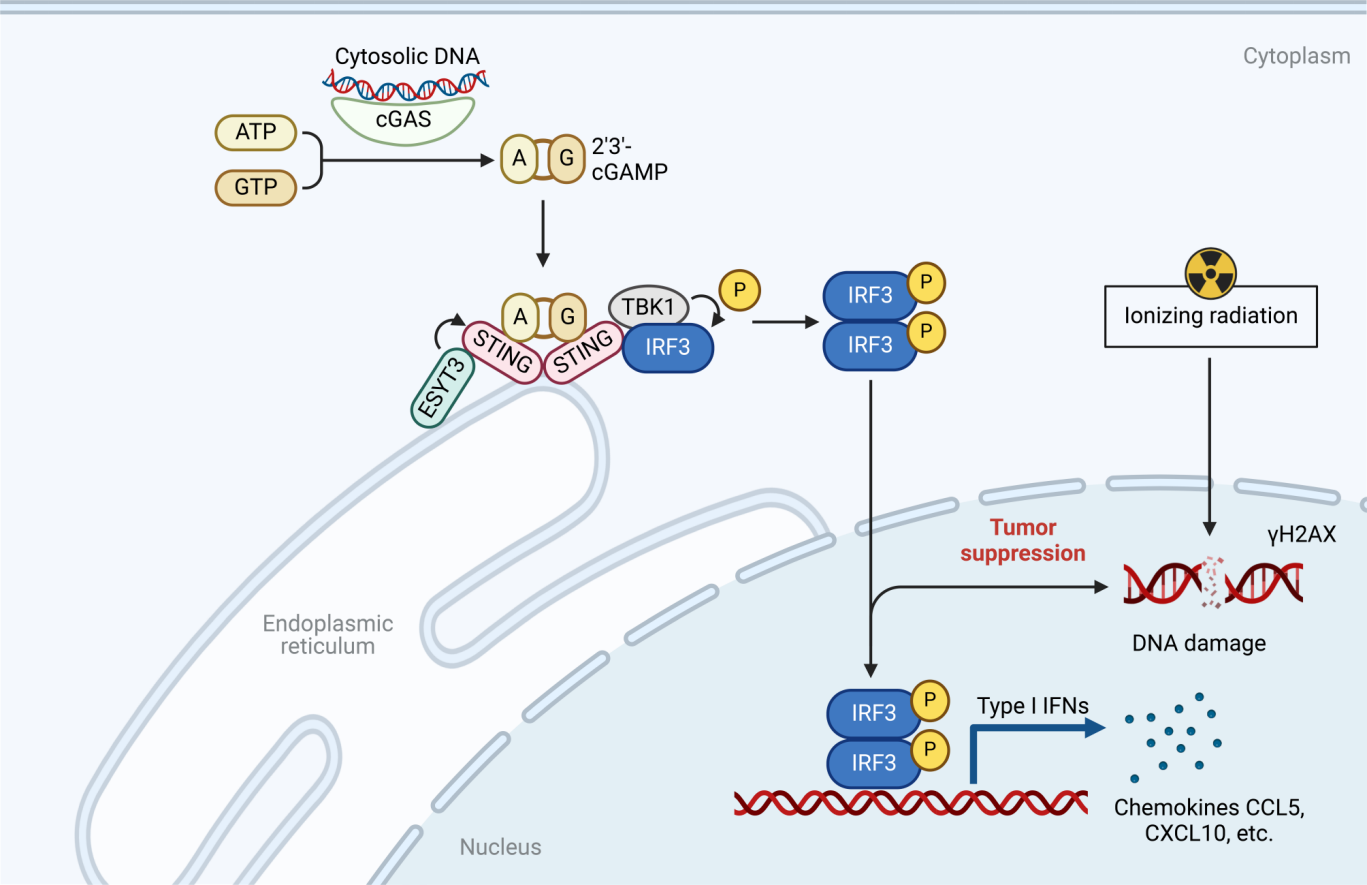
Schematic illustration of ESYT3 as a tumor suppressor and a novel radioimmune response sensitizer. ESYT3 directly interacted with STING, activated cGAS-STING pathway, and increased the generation of type I IFNs and downstream chemokines CCL5 and CXCL10, eventually enhancing radioimmune responses

To assess the combined treatment efficacy of ESYT3 overexpression and radiotherapy, we constructed xenograft tumor models. BALB/c nude mice were inoculated with ESYT3-overexpressing (OE-ESYT3) or control luciferase-tagged A549 cells. When the tumor reached ~ 50 mm^3^, the mice in the irradiation group or irradiation + OE-ESYT3 group received 5 Gy irradiation twice every 6 days. Overexpression of ESYT3 in the combination of irradiation displayed a good effect in hindering in vivo tumor growth (Fig. 7A-D). Additionally, IFNβ, CCL5 and CXCL10 levels were prominently attenuated by ESYT3 overexpression combined with irradiation (Fig. 7E-H), further demonstrating the synergetic effect in inducing radioimmune responses. To further analyze the influence of the combination therapy on immune response, a syngeneic mouse model was established: C57BL/6 mice were inoculated with OE-ESYT3 or control LLC cells and received irradiation treatment (Fig. 7I). The results showed that both ESYT3 overexpression and irradiation treatment improved the tumor-infiltrating levels of immune cells (CD3^+^CD8^+^ T cells and CD3^−^CD56^+^ NK cells) in the syngeneic mouse model, with a synergetic effect when combined the two (Fig. 7J-L). In conclusion, ESYT3 was identified as a tumor suppressor and a novel radioimmune response sensitizer, which directly interacted with STING, and activated cGAS-STING pathway, subsequently enhancing the generation of type I IFNs and downstream chemokines CCL5 and CXCL10, thus improving radioimmune responses (Fig. 8).

## Discussion

Radioresistance and unsatisfactory efficacy of radioimmunotherapy remains critical challenges to LUAD therapy [36]. This work proposed a novel classification of TCGA-LUAD patients into radioresistant subtype (C1) or radiosensitive subtype (C2) on the basis of prognostic DERRGs. C1 subtype exhibited poorer OS outcomes and more advanced stage in comparison to C2 subtype. Most RRGs that were overexpressed in radioresistant LUAD cells displayed the notable up-regulation in C1, with opposite results in C2, which demonstrated C1 as radioresistant subtype as well as C2 as radiosensitive subtype. The reliability and repeatability of radioresistant and radiosensitive subtypes were proven in multiple cohorts.

In accordance with the reduced ROS level, higher sensitivity to responses to UV and X-ray as well as increased activity of most DNA damage repair pathways, C1 subtype was radioresistant. Cellular responses to DNA damage represent critical determinants of tumor development and clinical outcomes following radiotherapy. Despite the connections of dysregulated DNA damage response with predisposition to tumor development, DNA damage response also induces hypersensitivity or resistance, which may be utilized for improving cancer therapy [37]. More frequent somatic mutations as well as copy-number amplifications and deletions occurred in C1 versus C2 subtype. Previous evidence proves that *STK11/LKB1* mutations in LUAD correlate to KEAP1/NRF2-dependent radioresistance targetable by glutaminase suppression [38, 39]. Ferroptosis inactivation occurred in C1 radioresistant subtype. Evidence suggests that radiotherapy-induced lipid peroxidation contributes to ferroptosis and radiotherapy can synergize with ferroptosis inducers [40, 41]. In addition, targetable CoQ-FSP1 signaling triggers ferroptosis- and radiotherapy-resistance in KEAP1-inactive LUAD [42]. ICB has prolonged the survival of patients with advanced LUAD, but such therapeutic efficacy is still unsatisfactory because most patients do not have an objective response and initially develop primary or acquire resistance soon following treatment [43–45]. Consequently, many approaches for improving the systemic efficacy of ICB have been proposed, notably combination of radiotherapy with ICB [46–48]. It was also demonstrated the low infiltration of most immune cells in C1 subtype, unveiling the immunosuppressive features in this radioresistant subtype. Based upon TIDE prediction, C2 subtype patients potentially responded to immunotherapy. Combination of radiotherapy with ICB might be effective for LUAD patients.

Machine learning has been extensively applied in the field of precision oncology [49]. To quantify the radioresistant and radiosensitive subtypes, we proposed the RRscore system, which enabled to predict LUAD prognosis in many independent cohorts. A meta-analysis showed that patients show significant immunological alterations within one-month radiotherapy, which result in T lymphocyte apoptosis and decrease, and influence peripheral blood immune cell balance [50]. Neutrophils remain the most abundant circulating leucocyte type, which are critical for innate immune response. Pro- or antitumoral features are attributed to tumor-associated neutrophils, macrophage and other immune cells in tumor microenvironment [51–53]. LUAD growth and radioresistance depend upon GLUT1-induced glucose uptake in tumor-associated neutrophils [54]. Our work demonstrated the strongly positive connections of the RRscore with activated CD4^+^ T cells and neutrophils across LUAD patients. Based upon the immunotherapy cohort, patients with low RRscore presented the higher possibility of responding to anti-PD-L1 blockade. Despite this, the efficacy of the RRscore system in predicting response to ICB requires to be proven in larger LUAD immunotherapy cohorts.

The RRscore was composed of FAM83A, PLEK2, PTPRH, BEX4, ESYT3 and LYPD3. It was proven that PTPRH, BEX4, and LYPD3 were overexpressed while FAM83A, PLEK2, and ESYT3 were down-regulated both in radioresistant LUAD tissues and cells. Among them, FAM83A has been proven as a prognostic biomarker as well as associates with tumor-infiltrating lymphocytes in smoking-related LUAD [55]. FAM83A up-regulates PD-L1 expression through ERK pathway, and FAM83A/PD-L1 co-expression contributes to undesirable survival of LUAD patients [56]. Up-regulated PLEK2 enables to independently predict worse progression-free survival in LUAD [57]. PTPRH overexpression also associates with poor patient survival [58]. mTOR up-regulation of BEX4 triggers LUAD cellular proliferation through potentiating OCT4 [59]. High LYPD3 expression correlates to unfavorable LUAD survival [60]. Overall, combining previous research, these genes exert crucial functions in LUAD. ESYT3 function as a Ca^2+^-regulated intrinsic membrane protein. Low expression of ESYT3 is associated with immune cell infiltration and poor prognosis in LUAD [35]. Our experiments proved that ESYT3 overexpression enabled to attenuate LUAD radioresistance and improve radiosensitivity. We discovered ESYT3 as a tumor suppressor as well as a novel radioimmune response sensitizer. ESYT3 enabled to activate cGAS-STING-dependent DNA damage response, subsequently enhancing the generation of type I IFNs and downstream chemokines CCL5 and CXCL10, eventually improving radioimmune responses. More importantly, the combination treatment of ESYT3 overexpression with radiotherapy exerted a synergistic anti-cancer effect, providing a possible combination treatment strategy against LUAD. cGAS-STING pathway senses self-DNA derived from damaged and dying cells and induces antitumor immunity by activating type I IFN [61]. The combination of STING agonist with immune checkpoint inhibitor could simultaneously boost the innate and adaptive immunity in preclinic study and acquire synergistic antitumor response in clinic trial [62–64]. Thus, we predict that low expression of ESYT3 may also attribute to immunotherapy resistance of LUAD.

## Conclusion

Altogether, this work innovatively proposed a radiotherapy response classification for LUAD, and discovered ESYT3 acted as a tumor suppressor and a novel radioimmune response sensitizer. Mechanically, ESYT3 alleviated LUAD radioresistance through activation of cGAS-STING-dependent pathway. Overall, our findings provide the explanation for the molecular mechanisms underlying radioimmune responses, and uncover the promising combination treatment of ESYT3 overexpression with radiotherapy for LUAD.

### Electronic supplementary material

Below is the link to the electronic supplementary material.

Supplementary Material 1

Supplementary Material 2

Supplementary Material 3

Supplementary Material 4

Supplementary Material 5

Supplementary Material 6

Supplementary Material 7

Supplementary Material 8

Supplementary Material 9

Supplementary Material 10

Supplementary Material 11

Supplementary Material 12

Supplementary Material 13

Supplementary Material 14

Supplementary Material 15

Supplementary Material 16

Supplementary Material 17

## Data Availability

No datasets were generated or analysed during the current study.

## Abbreviations

NSCLC: Non-small cell lung cancer
LUAD: Lung adenocarcinoma
PD-1: Programmed death 1
PD-L1: Programmed death ligand 1
RRGs: Radioresistance-related genes
DEGs: Differentially expressed genes
DERRGs: Differentially expressed RRGs
FC: Fold change
SNV: Single nucleotide variation
CNV: Copy number variation
GSCA: Gene Set Cancer Analysis
TMB: Tumor mutation burden
PCA: Principal component analysis
OS: Overall survival
ROS: Reactive oxygen species
MSigDB: Molecular Signatures Database
ssGSEA: Single-sample gene set enrichment analysis
NTP: Nearest template prediction
ICD: Immunogenic cell death
TIDE: Tumor Immune Dysfunction and Exclusion
CR: Complete response
PR: Partial response
SD: Stable disease
PD: Progressive disease
RRscore: Radioresistance score
LASSO: Least absolute shrinkage and selection operator
ROC: Receiver operator characteristic curve
DFS: Disease-free survival
ICB: Immune checkpoint blockade
IF: Immunofluorescence
IHC: immunohistochemistry
Co-IP: Coimmunoprecipitation
gDNA: Genomic DNA
mtDNA: Mitochondrial DNA
