# Supplementary material for: Overexpression of ESYT3 improves radioimmune responses through activating cGAS-STING pathway in lung adenocarcinoma
Source: Exp Hematol Oncol. 2024 Aug 5;13:77. doi: 10.1186/s40164-024-00546-y (PMC11302107; doi:10.1186/s40164-024-00546-y)
Supplement: Supplementary file 1 — Supplementary Material 1 [file 40164_2024_546_MOESM1_ESM.docx]

**Supplementary files:**

Supplementary figure 1. Classification of LUAD patients into radioresistant and radiosensitive subtypes. (A, B) Selection of DEGs in LUAD versus control specimens under the criteria of |log2FC|>1 and adjusted p<0.05. (C) Venn chart of the DERRGs via intersecting the DEGs and RRGs. (D) Consensus matrix at k=2 based upon the transcriptome profiling of the DERRGs. (E) The OS probabilities of radioresistant and radiosensitive subtypes. (F) Heatmap of the transcript levels of the DERRGs in two subtypes. (G) PCA for the transcriptome difference between subtypes. (H) The stage difference between two subtypes. (I-K) Comparison of the enrichment levels of (I) ROS, (J) X-ray, and UV responses, and (K) DNA damage repair pathways between subtypes. (L, M) The expression differences in ferroptosis, and ICD genes between subtypes. (N) Comparison of the abundance of immune components between subtypes. (O-Q) The differences in immune and stromal scores, and tumor purity between subtypes. (R-T) The differences in dysfunction, exclusion, and TIDE scores between subtypes. *p<0.05; **p<0.01; ***p<0.001.

Supplementary figure 2. Genetic mutation landscape of prognostic DERRGs across LUAD. (A) Waterfall plot illustrating the top ten mutated DERRGs. (B, C) Bar charts of the top ten copy-number amplified and deleted DERRGs, respectively.

Supplementary figure 3. The heterogeneity in genetic mutations and tumor immunity between the radioresistant and radiosensitive subtypes. (A-D) Copy-number amplifications (red) and deletions (blue) in (A, B) C1 and (C, D) C2 subtype. (E, F) Waterfall plots depicting the somatic mutations in two subtypes. (G) Comparison of TMB score between subtypes. ***p<0.001.

Supplementary figure 4. Assessment of the reliability and repeatability of the radioresistant and radiosensitive subtype classification in two LUAD external cohorts. (A) Heatmap of the top 100 up-regulated marker genes in C1 or C2 subtype in the GSE31210 dataset. (B, C) Verification of the patient classification, and OS difference between subtypes via NTP method in the GSE31210 dataset. (D) Heatmap of the top 100 up-regulated marker genes in C1 or C2 subtype in the GSE72094 dataset. (E, F) Validation of the patient classification, and OS difference utilizing NTP approach in the GSE72094 dataset.

Supplementary figure 5. Generation of a RRscore system for quantifying the radiotherapeutic response classification in LUAD. (A) Selection of lambda value via 10-fold cross-validation. (B) Coefficients of prognostic DERRGs in the LASSO analysis. (C) The distribution of RRscore, survival time and transcript levels of the identified genes. (D) ROCs at 1-, 3- or 5-year survival. (E) The OS probability of low or high RRscore patients. (F, G) Uni or multivariate-cox regression results on RRscore and clinical parameters with LUAD prognosis. (H) The nomogram composed of RRscore and stage for prognostication. (I) Comparison of the nomogram-estimated with actual survival. (J) Scatter plots demonstrating the relationships of the RRscore with radiotherapy response mechanisms: X-ray, and UV responses, and ROS. (K) The interactions of the RRscore with DNA damage repair pathways. (L, M) The connections of the RRscore with the transcript levels of immune checkpoints, and ICD molecules across TCGA-LUAD. (N) The relationships between the RRscore and the infiltration levels of immune components across TCGA-LUAD. (O) The percentages of distinct responses to anti-PD-L1 therapy in low- or high-RRscore individuals in the IMvigor210 cohort. (P) The OS probability of low- or high-RRscore individuals in the IMvigor210 cohort. (Q) Comparison of the RRscore between non-responders and responders in the IMvigor210 cohort. (R-T) Comparison of dysfunction, exclusion, and TIDE scores between low- and high-RRscore TCGA-LUAD patients. ***p<0.001; ns: p>0.05.

Supplementary figure 6. External verification of the reliability of the RRscore system in survival prediction. (A) The distribution of RRscore, survival duration as well as transcript levels of the DERRGs in the GSE31210 cohort. (B) The OS probability of low or high RRscore patients in the GSE31210 cohort. (C) ROC curves at 1-, 3-, or 5-year OS in the GSE31210 cohort. (D) The distribution of RRscore, survival duration, and transcript levels of the DERRGs in the GSE72094 cohort. (E, F) The OS probability of low or high RRscore patients as well as ROC curves at 1-, 3-, or 5-year OS in the GSE72094 cohort. (G) The DFS probability of low or high RRscore patients in the GSE30219 cohort. (H, I) The OS and ROC analysis in TCGA-LUAD patients who received radiotherapy.

Supplementary figure 7. ESYT3 expression is attenuated in LUAD radioresistance. (A-F) RT-qPCR analysis of the transcript levels of the prognostic DERRGs (PTPRH, BEX4, LYPD3, FAM83A, PLEK2, and ESYT3) in LUAD tissues with CR/PR or PD/SD. (G, H) Cell survival of radiosensitive or radioresistant LUAD cells upon distinct dosages of irradiation. (I) Representative RT-PCR images of the prognostic DERRGs (PTPRH, BEX4 and LYPD3) in radiosensitive and radioresistant LUAD cells. (J-L) RT-qPCR analysis of the expression of PTPRH, BEX4 and LYPD3 in radiosensitive and radioresistant LUAD cells. (M) Representative RT-PCR images of the prognostic DERRGs (FAM83A, PLEK2, and ESYT3) in radiosensitive and radioresistant LUAD cells. (N-P) RT-qPCR analysis of the expression of FAM83A, PLEK2, and ESYT3 in radiosensitive and radioresistant LUAD cells. *p<0.05; **p<0.01; ***p<0.001; ****p<0.0001.

Supplementary figure 8. ESYT3 acts as a potential tumor suppressor in LUAD. (A) ESYT3 expression in TCGA-LUAD and normal tissues. (B) Difference in ESYT3 expression among diverse pathological stages. (C, D) Analysis of OS and DFS in patients with low and high ESYT3 expression. (E) IHC staining of ESYT3 expression in representative human LUAD tissues (n=8). Bar, 200 μm. *p<0.05.

Supplementary figure 9. RT-PCR analysis results. (A) RT-PCR of ESYT3, IFNβ, CCL5 and CXCL10 in A549 and H1975 cells transfected with ESYT3 overexpression plasmids. (B, C) RT-PCR of IFNβ, CCL5 and CXCL10 in A549 and H1975 cells overexpressing ESYT3 following irradiation induction (5 Gy) for 2 hours.

Supplementary figure 10. Molecular docking of ESYT3 and STING.
