# Supplementary figures and images for: Overexpression of ESYT3 improves radioimmune responses through activating cGAS-STING pathway in lung adenocarcinoma
Source: Exp Hematol Oncol. 2024 Aug 5;13:77. doi: 10.1186/s40164-024-00546-y (PMC11302107; doi:10.1186/s40164-024-00546-y)

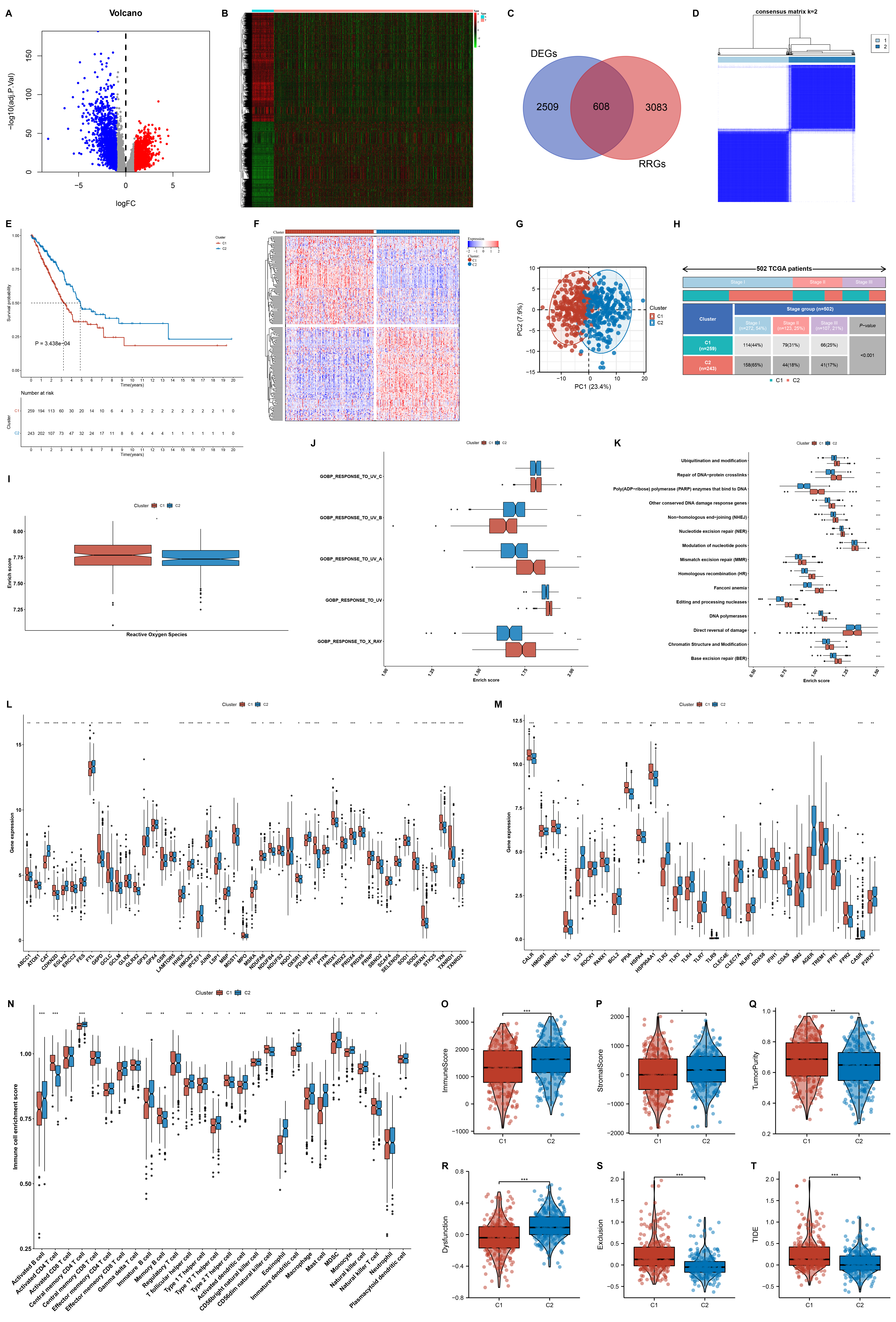

Supplement: Supplementary file 2 — Supplementary Material 2 [file 40164_2024_546_MOESM2_ESM.tif]

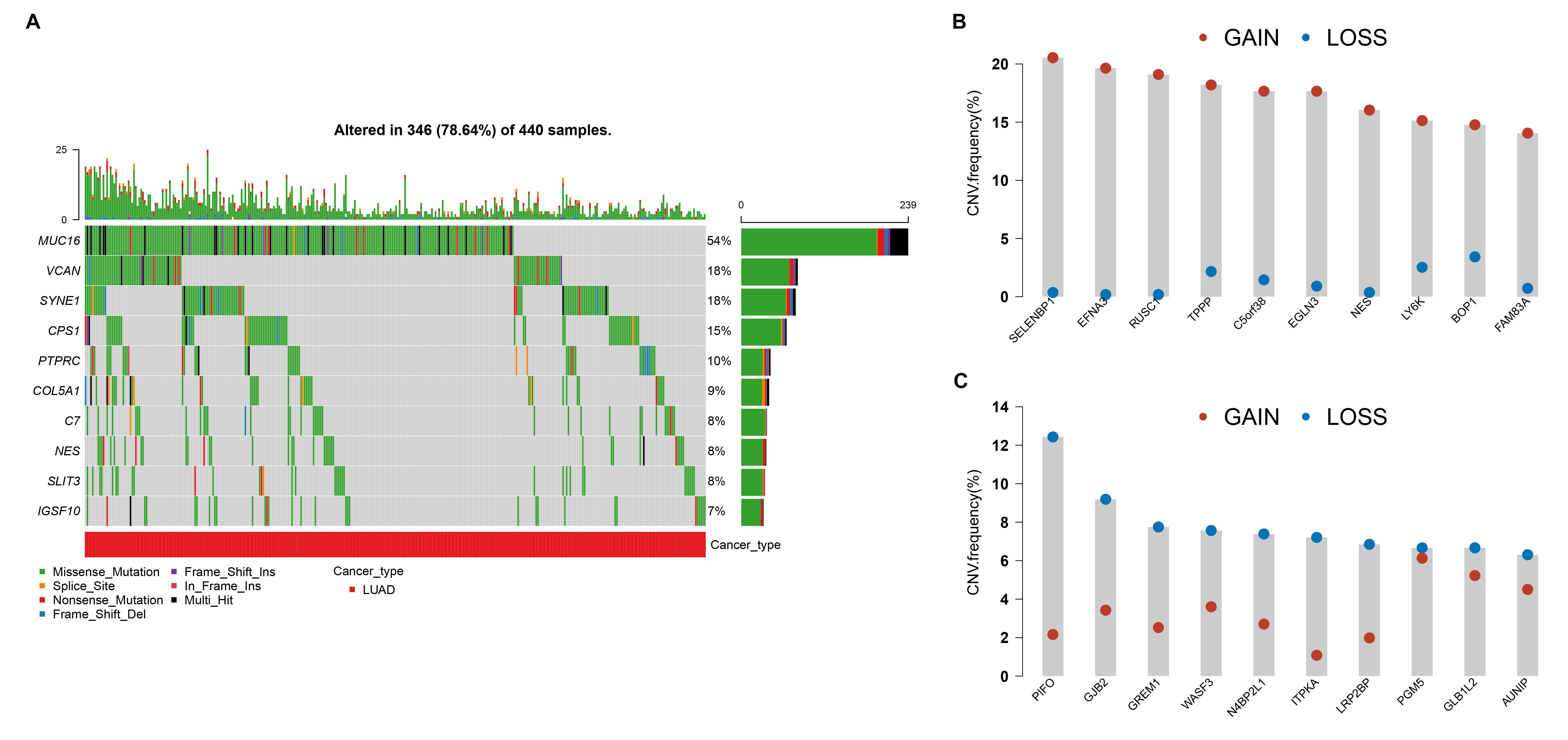

Supplement: Supplementary file 3 — Supplementary Material 3 [file 40164_2024_546_MOESM3_ESM.tif]

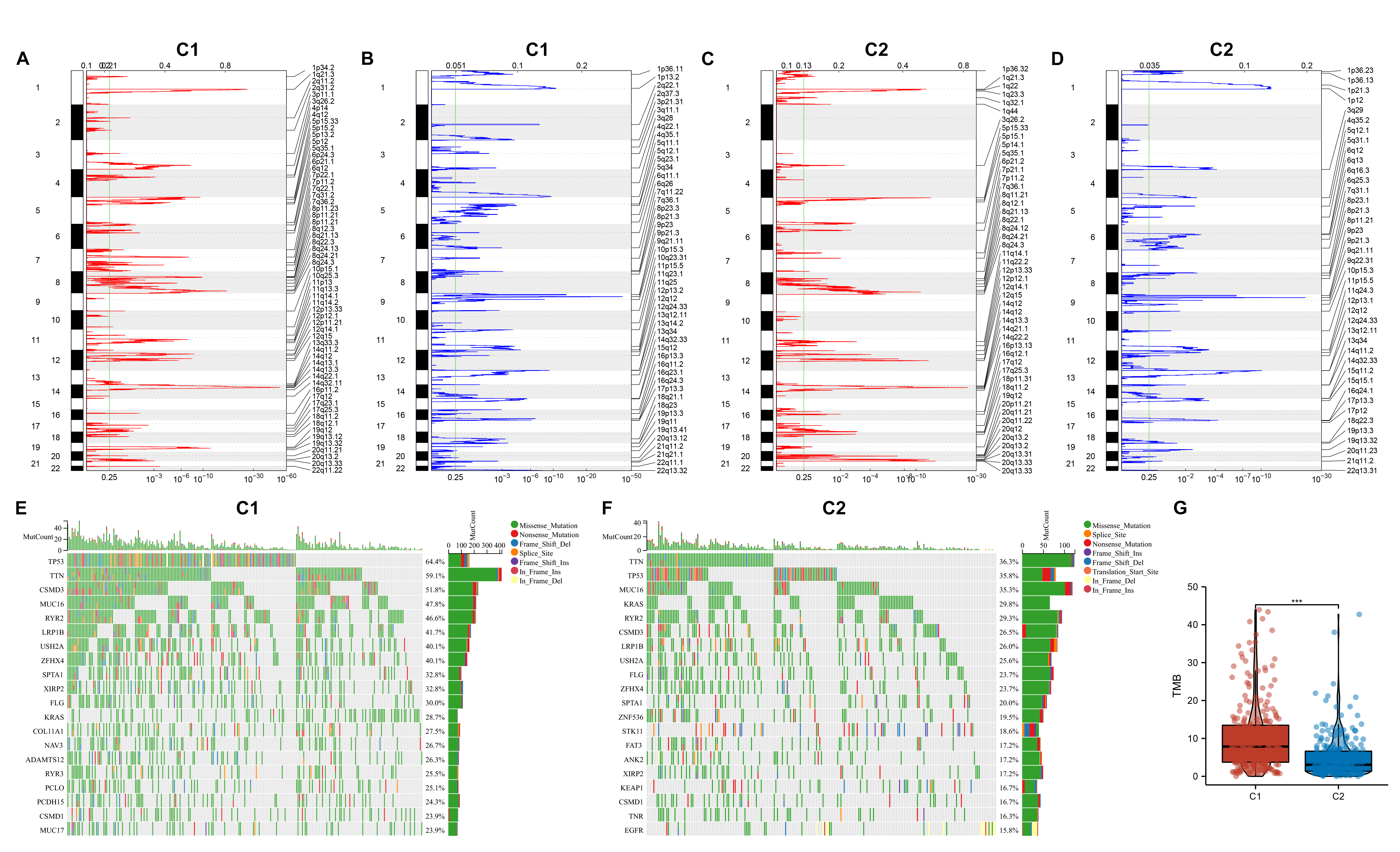

Supplement: Supplementary file 4 — Supplementary Material 4 [file 40164_2024_546_MOESM4_ESM.tif]

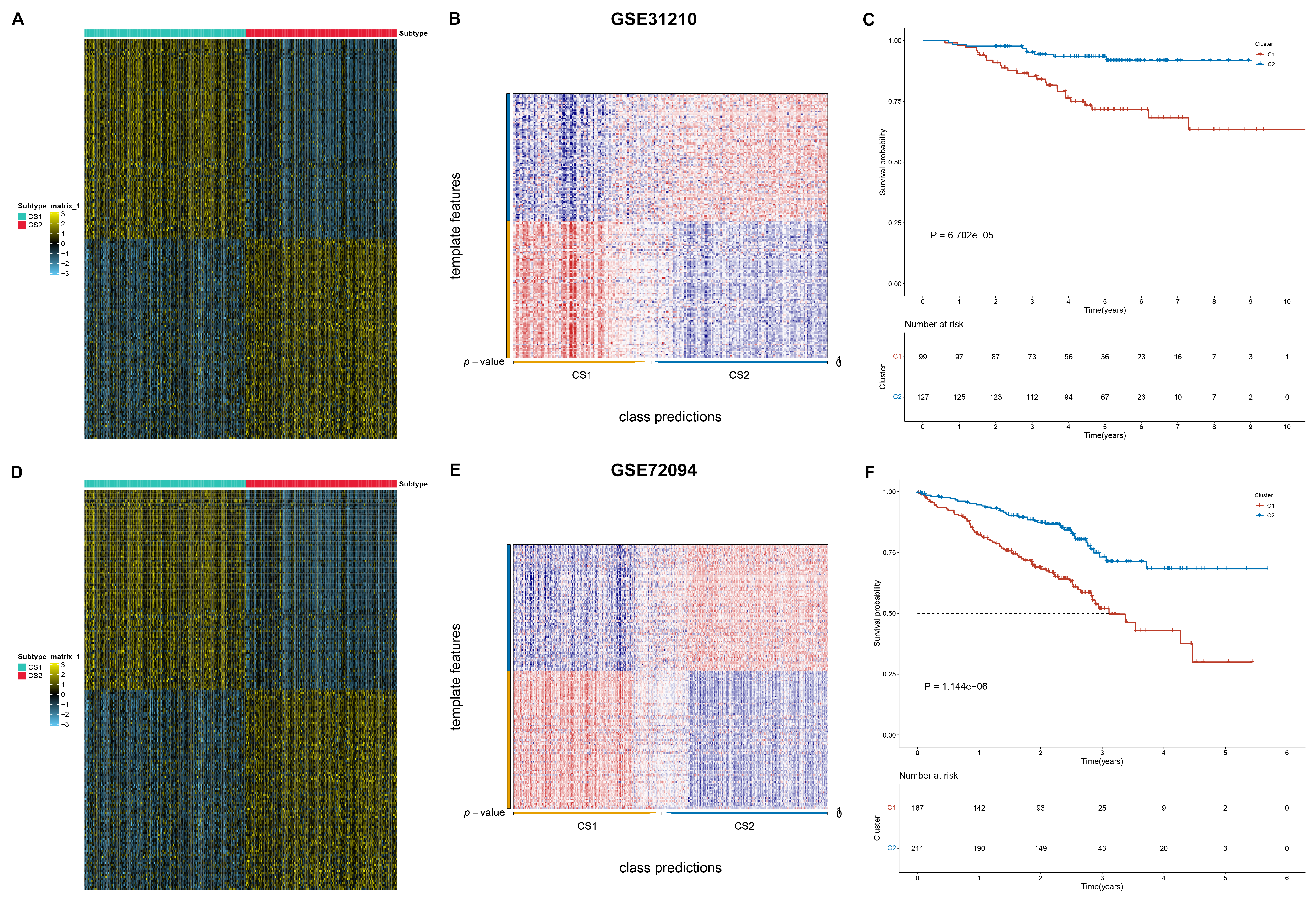

Supplement: Supplementary file 5 — Supplementary Material 5 [file 40164_2024_546_MOESM5_ESM.tif]

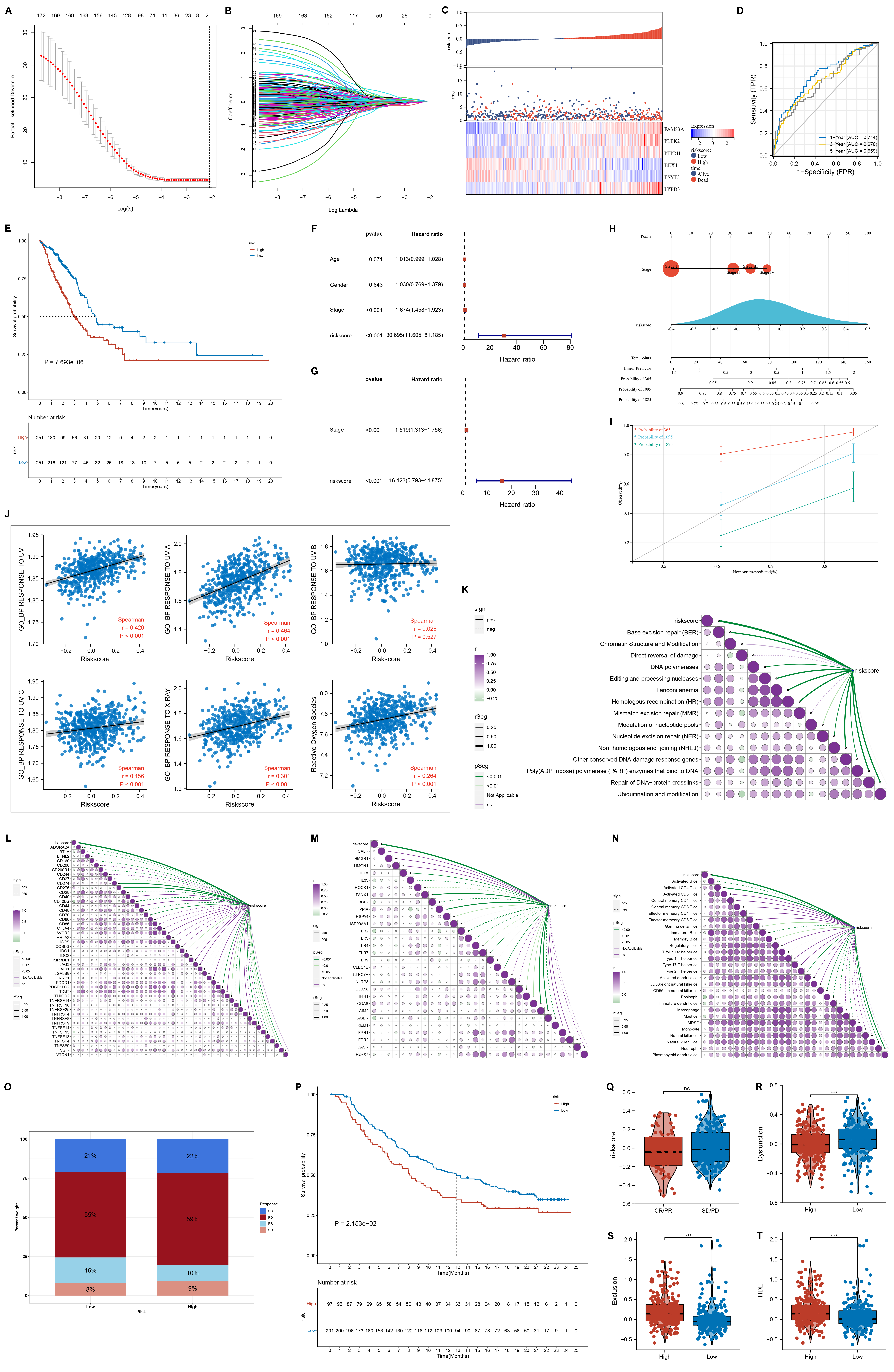

Supplement: Supplementary file 6 — Supplementary Material 6 [file 40164_2024_546_MOESM6_ESM.tif]

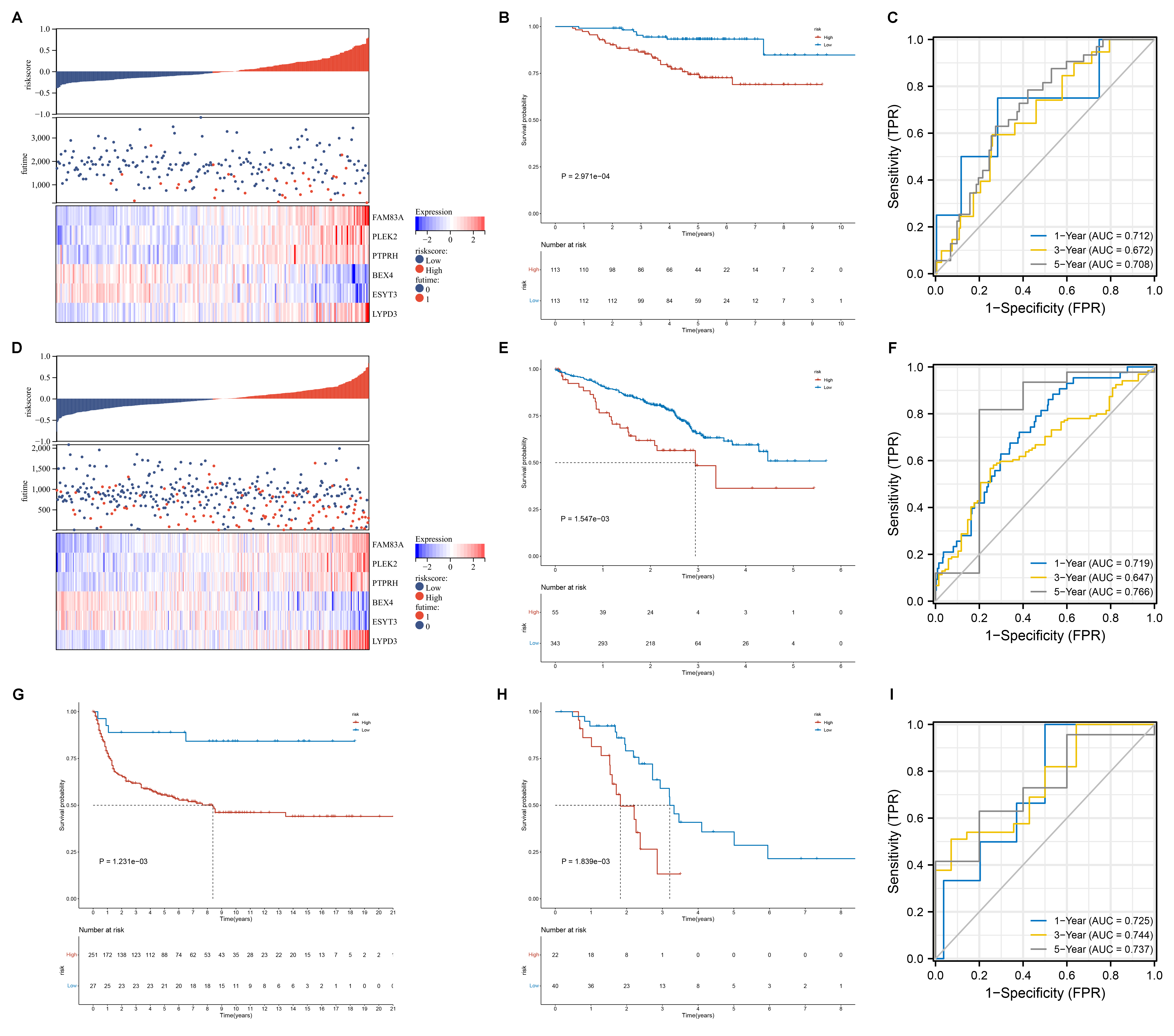

Supplement: Supplementary file 7 — Supplementary Material 7 [file 40164_2024_546_MOESM7_ESM.tif]

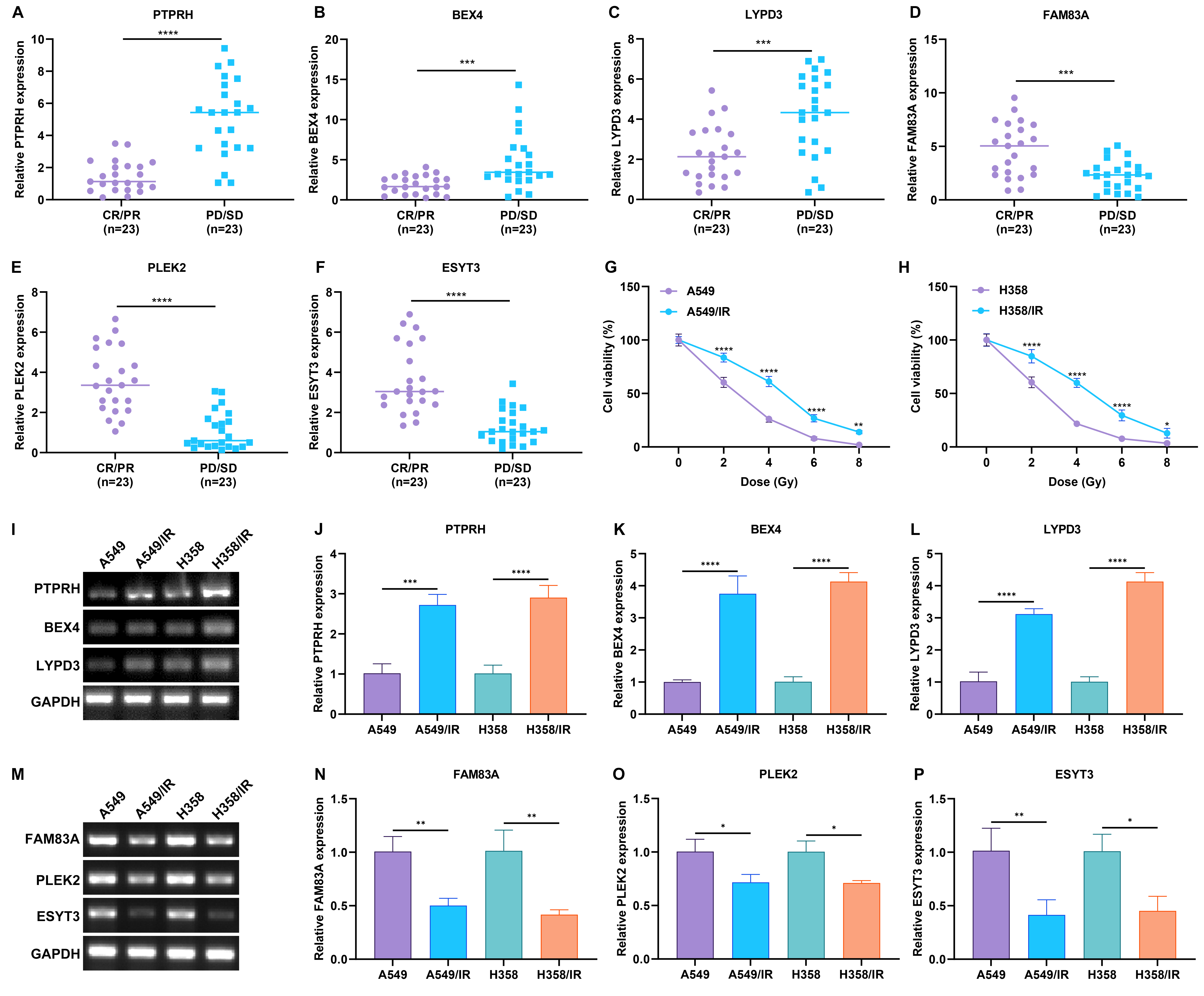

Supplement: Supplementary file 8 — Supplementary Material 8 [file 40164_2024_546_MOESM8_ESM.tif]

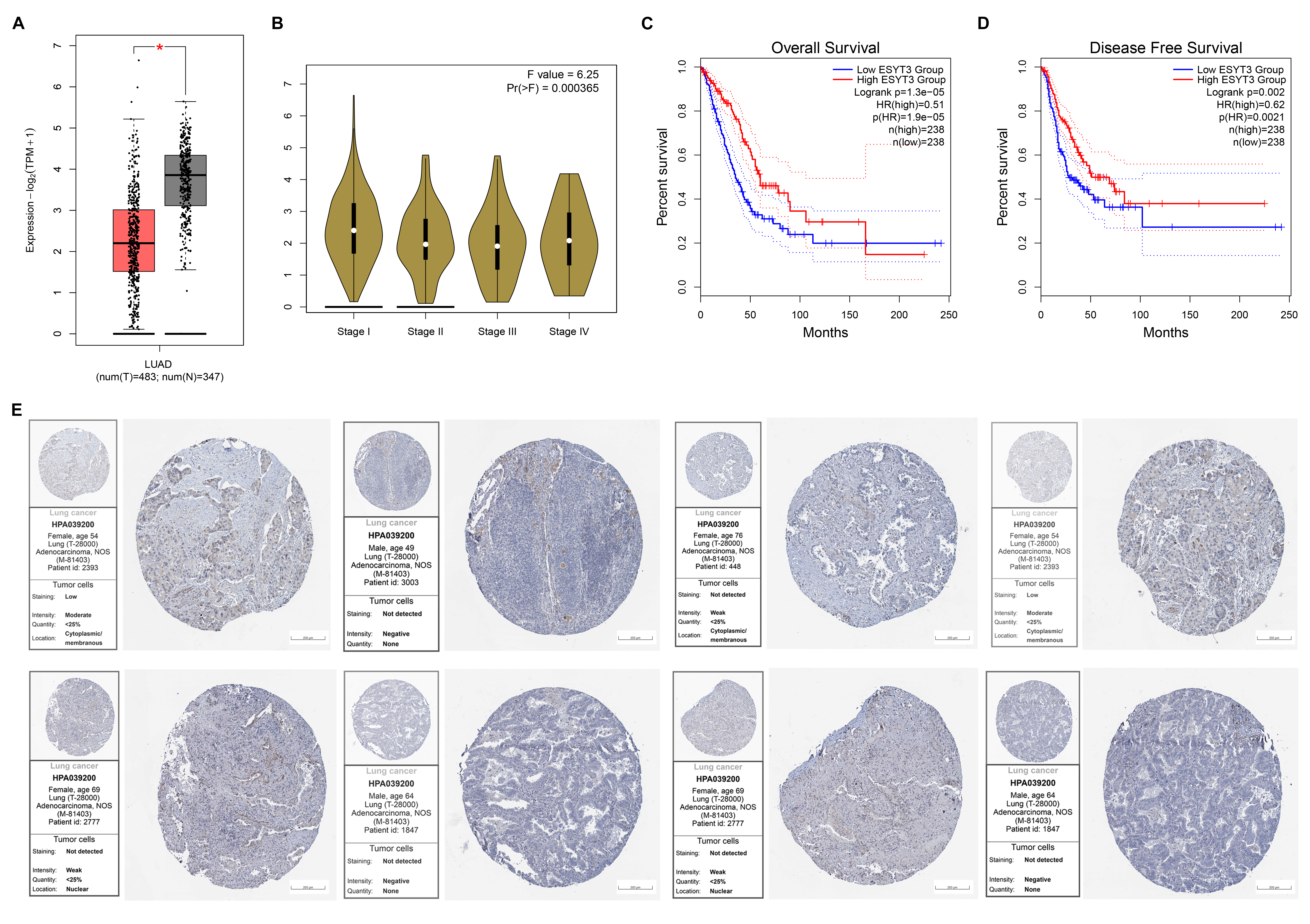

Supplement: Supplementary file 9 — Supplementary Material 9 [file 40164_2024_546_MOESM9_ESM.tif]

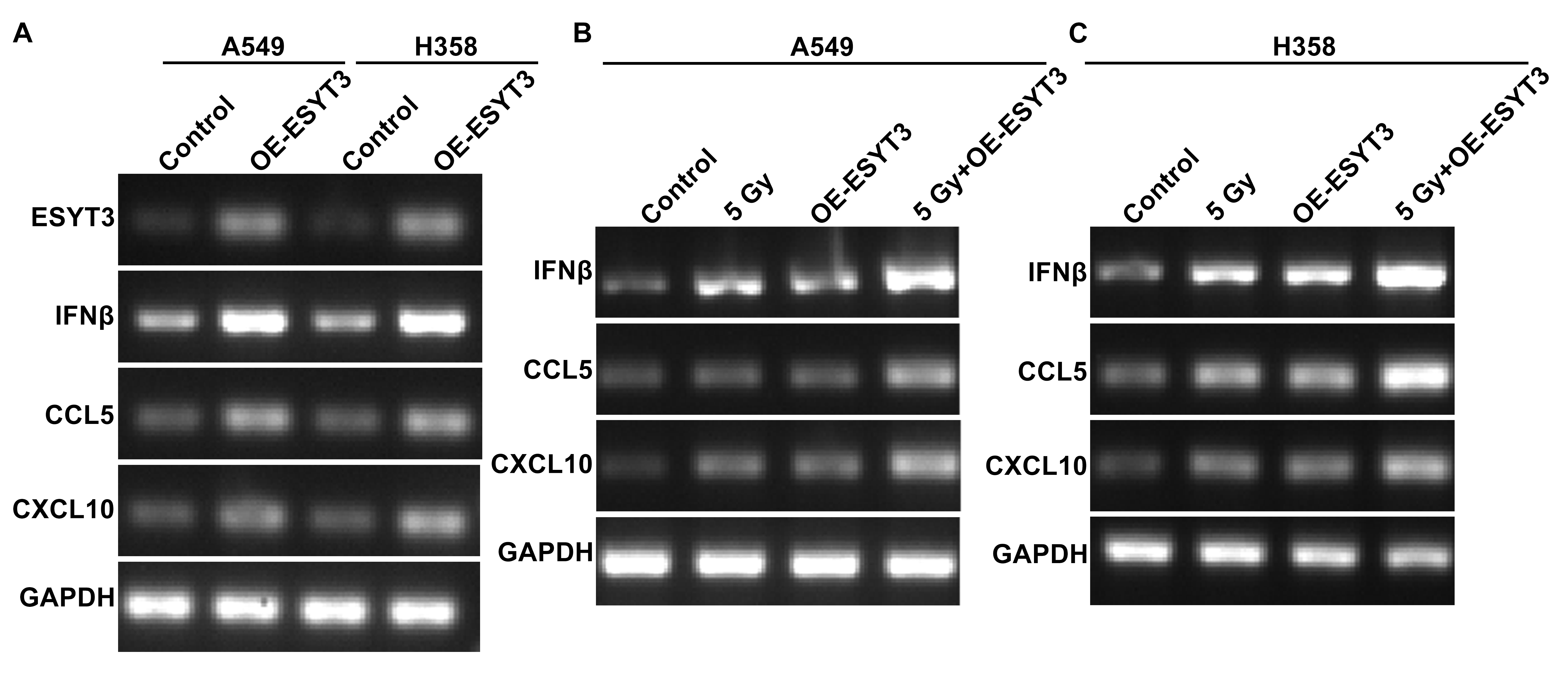

Supplement: Supplementary file 10 — Supplementary Material 10 [file 40164_2024_546_MOESM10_ESM.tif]

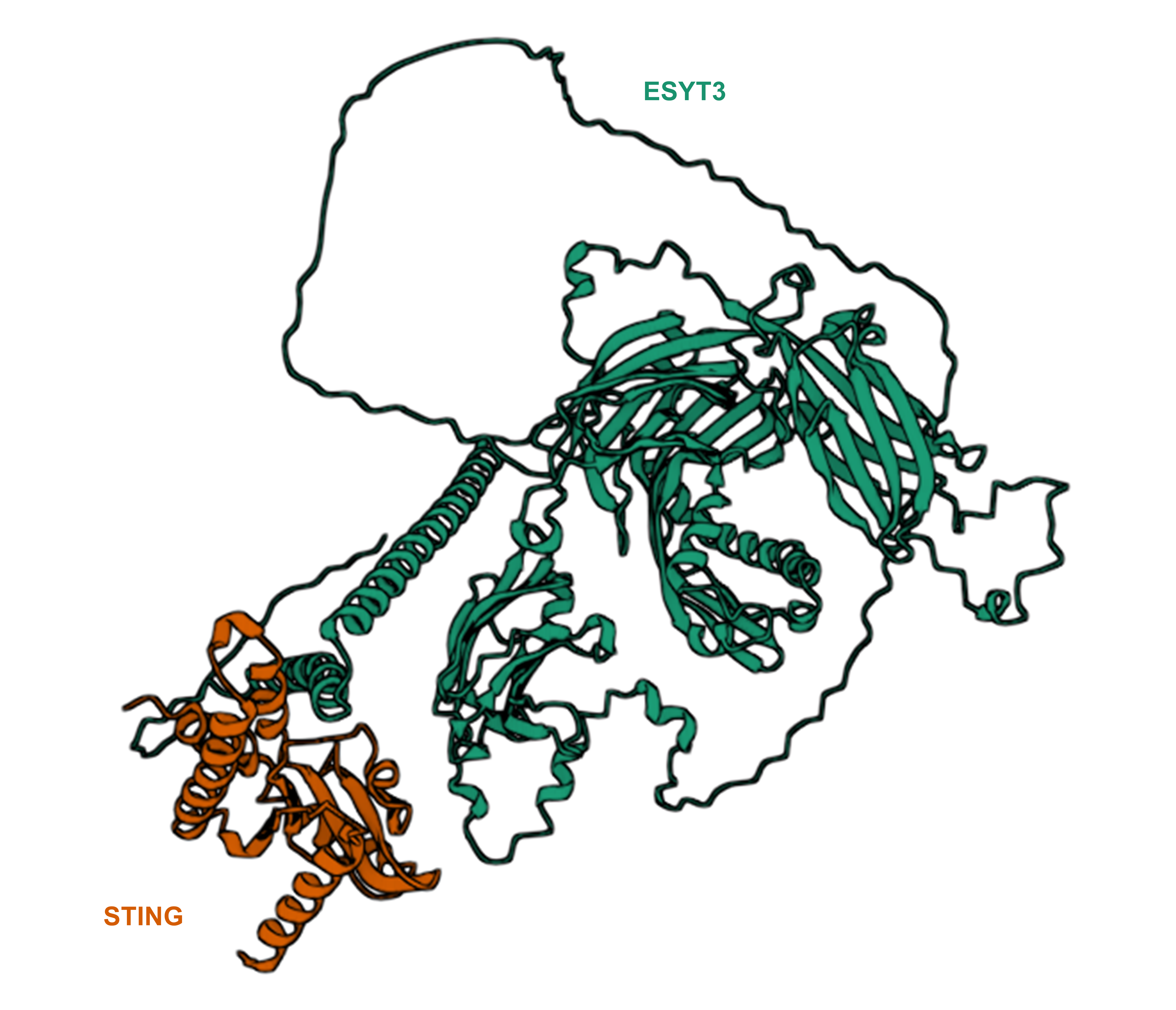

Supplement: Supplementary file 11 — Supplementary Material 11 [file 40164_2024_546_MOESM11_ESM.tif]
